# Myo19 Ensures Symmetric Partitioning of Mitochondria and Coupling of Mitochondrial Segregation to Cell Division

**DOI:** 10.1016/j.cub.2014.09.045

**Published:** 2014-11-03

**Authors:** Jennifer L. Rohn, Jigna V. Patel, Beate Neumann, Jutta Bulkescher, Nunu Mchedlishvili, Rachel C. McMullan, Omar A. Quintero, Jan Ellenberg, Buzz Baum

**Affiliations:** 1 Centre for Clinical Science and Technology, Division of Medicine, University College London, Wolfson House, London NW1 2HE, UK; 2 MRC Laboratory for Molecular Cell Biology, University College London, London WC1E 6BT, UK; 3 Advanced Light Microscopy Facility, European Molecular Biology Laboratory (EMBL), Meyerhofstrasse 1, 69117 Heidelberg, Germany; 4 Novo Nordisk Foundation Center for Protein Research, Blegdamsvej 3B, 2200 Copenhagen, Denmark; 5 Department of Biology, University of Richmond, Richmond, VA 23173, USA; 6 Department of Cell Biology and Biophysics, European Molecular Biology Laboratory (EMBL), Meyerhofstrasse 1, 69117 Heidelberg, Germany

## Abstract

During animal cell division, an actin-based ring cleaves the cell into two. Problems with this process can cause chromosome missegregation and defects in cytoplasmic inheritance and the partitioning of organelles, which in turn are associated with human diseases [1, 2, 3]. Although much is known about how chromosome segregation is coupled to cell division, the way organelles coordinate their inheritance during partitioning to daughter cells is less well understood. Here, using a high-content live-imaging small interfering RNA screen, we identify Myosin-XIX (Myo19) as a novel regulator of cell division. Previously, this actin-based motor was shown to control the interphase movement of mitochondria [4]. Our analysis shows that Myo19 is indeed localized to mitochondria and that its silencing leads to defects in the distribution of mitochondria within cells and in mitochondrial partitioning at division. Furthermore, many Myo19 RNAi cells undergo stochastic division failure—a phenotype that can be mimicked using a treatment that blocks mitochondrial fission and rescued by decreasing mitochondrial fusion, implying that mitochondria can physically interfere with cytokinesis. Strikingly, using live imaging we also observe the inappropriate movement of mitochondria to the poles of spindles in cells depleted for Myo19 as they enter anaphase. Since this phenocopies the results of an acute loss of actin filaments in anaphase, these data support a model whereby the Myo19 actin-based motor helps to control mitochondrial movement to ensure their faithful segregation during division. The presence of DNA within mitochondria makes their inheritance an especially important aspect of symmetrical cell division.

## Highlights

• RNAi screen identifies Myo19 as novel regulator of cell division
• Myo19 ensures fair mitochondrial inheritance at division
• Division requires coupled mitochondrial segregation and cytokinesis

## Teaser

It is not known how animal cells partition mitochondria at division. Rohn et al. identify the unconventional myosin Myo19 as a novel actin-based regulator of this process. Myo19 depletion alters the distribution of mitochondria at mitotic exit, leading to unequal mitochondrial segregation at division and, occasionally, to division failure.

## Results and Discussion

To ensure faithful organelle inheritance, the segregation of each cellular component must be tightly coupled to the act of cell division. For chromosomes, this coupling relies on the exchange of signals between the elongating anaphase spindle and the overlying cell cortex, which helps to position the site at which the actomyosin-based ring is formed that cuts the cell into two [5]. Although the mechanisms are less well worked out, organelles may also rely on crosstalk between the microtubule-based spindle and the actin cortex for their partitioning [6, 7, 8]. To identify new actin-based regulators of cell division, we screened a human “actinome” small interfering RNA (siRNA) library [9] for siRNAs that induce division errors, targeting genes associated with the actin cytoskeleton, genes with predicted actin-binding domains, myosin motors, Rho family GTPases, GTPase activating proteins (GAPs), and guanine nucleotide exchange factors for siRNAs that induce division errors. While previous screens had used fixed endpoint assays to identify cytoskeletal regulators whose silencing led to cytokinesis failure (e.g., [10]), here we aimed to combine fixed data with live imaging to identify siRNAs that caused more subtle division errors.

Briefly, for the live-imaging analysis, a library targeting the human actinome, four siRNAs per gene, was mixed with a transfection reagent and arrayed in spots onto glass chamber slides [11]. HeLa-13 cells expressing LifeAct-EGFP to label filamentous actin and histone-2B-mCherry to label DNA [12] were then plated onto these arrays in triplicate experiments. Approximately 2 days after solid-phase reverse transfection, these marked islands of siRNA-treated cells were then filmed, using automated microscopy, to take a frame every 33 min over a 20 hr period. All images are freely available on our curated RNAi website FLIGHT.

**Figure 1:**
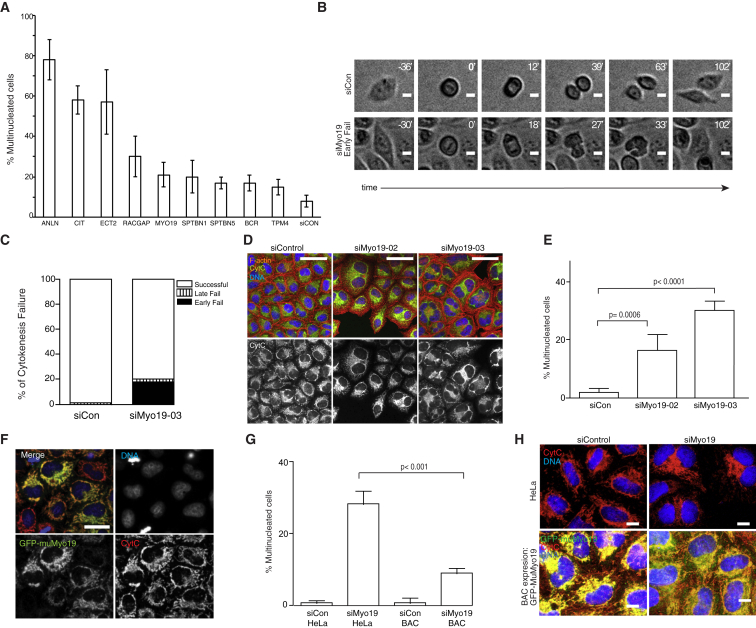
A Live-Image Screen Identifies a Role for Myo19 in Faithful Cell Division (A) Division failure resulting in a multinucleated HeLa cell during live imaging from day 2 to day 3 postsilencing of various genes is shown relative to the negative siControl. The screen was performed in triplicate with four independent siRNAs per gene. The pooled mean of each gene is shown; bars indicate SD. See Figure S1A for a summary of the workflow and Table S1 for the individual siRNA phenotypes. (B) Bright-field micrographs of cells live imaged every 3 min for 48 hr after silencing with the indicated siRNAs. Cell of interest is in middle of field; timestamp is in minutes, with zero set to the beginning of anaphase. Scale bars, 10 μm. (C) Quantification of two experiments similar to that shown in (B) indicating mean percentage (at least 100 cells were counted per experiment) of cells that failed cell division early (soon after furrow formation) or late (later on in cytokinesis). (D) Representative micrographs of HeLa cells silenced with the indicated siRNAs and fixed 65 hr posttransfection. DAPI staining (DNA) is blue, phalloidin (F-actin) is red, and cytochrome *c* is gray. Scale bars, 50 μm. (E) Quantification of three experiments similar to (D) (at least 100 cells were counted per experiment; bars are SD). (F) Micrographs of the HeLa BAC muMyo19 cell line, showing that the Myo19 division phenotype is rescued by a murine BAC copy of the gene. The grayscale images show individual channels as indicated; the color composite image shows DAPI staining (DNA; blue), cytochrome *c* (mitochondria; red), and GFP (muMyo19-GFP; green). Scale bar, 30 μm. (G) Gene silencing of Myo19 compared with control in strongly GFP-positive HeLa BAC muMyo19 cells versus nontransgenic HeLa cells, showing that the muMyo19 can rescue the multinucleated cell phenotype caused by Myo19 knockdown alone. Experiment was performed three times with triplicate wells; the mean and SD of percent multinucleated cells is shown. (H) Representative images from experiment performed in (G); same color channels as for (F). Scale bar, 10 μm. See Figures S1B and S1C for quantitation of the phenotype in an independent cell type and Figure S1D for quantitation of the siRNA silencing in HeLa cells by quantitative PCR. See also Figure S1 and Table S1.

We focused our manual screen analysis on hits (n = 67) that exhibited a multinucleated RNAi phenotype in the fixed screen carried out using the same library [9]. Movies were visually inspected to identify siRNAs inducing cell division defects. For the 18/67 hits with the most reproducible oligo-specific RNAi phenotypes, division outcome was scored for 100 cells in each film and was compared with the outcomes from siControl spots on the same slide. Using this approach, nine candidate genes were identified that exhibited a cell division defect with more than one independent siRNA (Figure 1A; for details of these siRNAs and their individual phenotypes, see Table S1 and see Figure S1A, available online, for a graphical depiction of the workflow).

The strongest hits corresponded to genes known to be crucial for faithful cell division, including Anillin [13], Citron kinase [14], and Ect2 [15]. The centralspindlin subunit Rac GTPase activating protein 1 (RACGAP1, MgcRacGAP) [16] was also identified as a moderately strong hit, together with two members of the beta-spectrin family [17], which bind actin and are major constituents of the cell cortex, and an unconventional myosin, Myosin-XIX (Myo19) [4]. Finally, BCR, which contains a C-terminal GAP domain specific for Rac [18], and TPM4 (tropomyosin 4 [19]) were recovered as relatively weak hits.

Given its potential novelty, we chose to focus our further analysis on the role of the unconventional myosin Myo19. Briefly, Myo19 is a myosin found in most animals [20] that appears to have been lost from lineages leading to insects and roundworms [21]. The 970 amino acid protein consists of a motor domain that has features distinguishing it from other myosin classes [22, 23, 24], a lever arm region containing three light-chain-binding IQ-motifs, and a tail domain. Interestingly, the tail domain of Myo19, which is unique in the myosin family, was recently shown to target the protein to mitochondria [4]. This *My*o19-specific *m*itochondria *o*uter *m*embrane *a*ssociation (MyMOMA) domain consists of approximately 150 amino acids (140 in mouse), bearing little easily identifiable sequence or structural homology to other known domains. Previous biochemical studies of purified Myo19 confirmed that the protein is a plus-end-directed [25], actin-activated ATPase capable of translocating actin filaments in vitro [22]. These studies also suggested that Myo19 may spend a large fraction of its chemomechanical cycle in an actin-bound state—a property common to transport motors such as Myo5 [26]. Moreover, the ectopic expression of full-length Myo19 was reported to increase the motility of mitochondria in interphase and to alter mitochondrial network organization in an actin-dependent fashion [4]. Given this, it was important to first confirm a role for Myo19 in division, before going on to test whether Myo19 might influence the outcome of cell division through its interaction with mitochondria.

### Myo19 Knockdown Leads to Cytokinesis Failure

Using a Myo19 siRNA (Myo19-03), we were able to confirm the increased rate of division failure following Myo19 silencing using live imaging (Figure 1B) and to show that in the majority of cases (37/41), division first failed soon after furrow formation, while only a small number of cells failed later (Figure 1C). To validate this Myo19 RNAi phenotype, we silenced the gene using nonoverlapping siRNAs. After fixation and staining, we observed a significant increase in the percentage of Myo19 RNAi cells with more than one nucleus compared with the nontargeting siControl (Figure 1D). Specifically, after 2 days of treatment with Myo19-02 siRNA, 14% of cells were multinucleated, whereas Myo19-03 siRNA caused 30% of the cells to contain multiple nuclei, compared to 2% of siControl-treated cells (Figure 1E). A similar phenotype was obtained using two additional siRNA sets targeting the same gene (data not shown).

To further validate the specificity of the Myo19 depletion phenotype, we performed a rescue experiment in a HeLa cell line stably expressing an N-terminally tagged GFP-bacterial artificial chromosome (BAC) version of murine Myo19, whose species-specific nucleotide sequence differences would make it resistant to knockdown by human Myo19 siRNA. In these cells, the entire visible pool of GFP-Myo19 was localized to mitochondria, as confirmed by colabeling with MitoTracker (data not shown) and by fixing and staining cells with an antibody against cytochrome *c* (Figure 1F). Moreover, in cells with robust mitochondrial GFP-Myo19 expression (Figure 1G, with representative images in Figure 1H), we observed a statistically significant reduction in the percentage of multinucleated cells following treatment with Myo19-03 siRNA, implying a direct or indirect role for this mitochondrially localized pool of Myo19 in cell division.

As additional validation, we observed a significant increase in the percentage of multinucleation in a different cell type, namely, murine cells expressing a stable, lentiviral-induced Myo19 small hairpin RNA (63% of silenced cells, compared with 34% in the control; Figure S1B), in which the Myo19 knockdown (of ∼80%) could be verified by western blot analysis using an antibody generated against the mouse protein (Figure S1C). We also used quantitative PCR to validate the knockdown following treatment with Myo19-02 and Myo-19-03 siRNAs in HeLa cells, revealing an 88% reduction with Myo19-02 and a 73% reduction with Myo19-03 oligo relative to the siControl reagent (Figure S1D). From this point on, we used Myo19-03 as the representative siRNA for our experiments, as it had the most penetrant phenotype.

### Defects in Mitochondrial Organization Can Interfere with Cell Division

**Figure 2:**
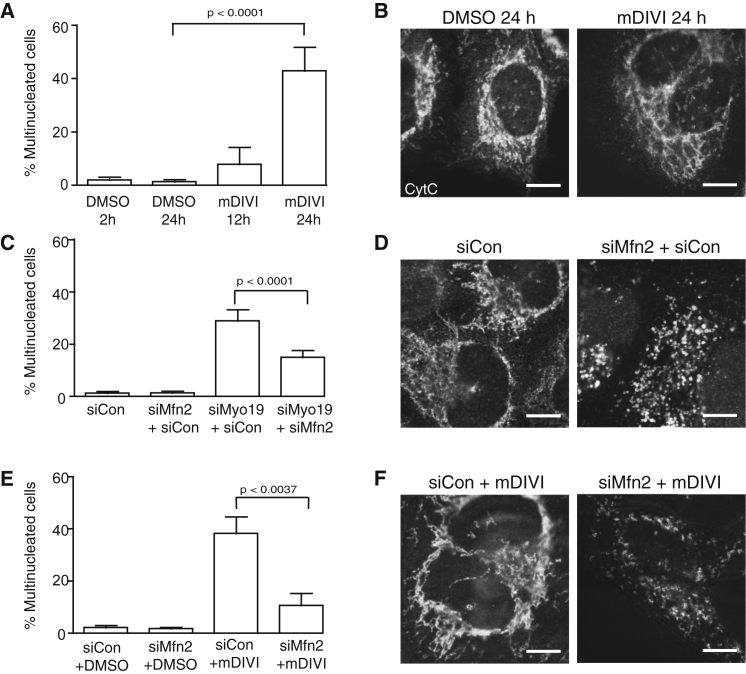
Inappropriate Mitochondrial Fusion Adversely Affects Cell Division, and Enhanced Fission Can Rescue the Myo19 Phenotype (A and B) Quantification of multinucleation in HeLa cells treated with 10 μM Mdivi (100 cells were counted; mean of five experiments for 12 or 24 hr), with (B) showing representative cells at 24 hr posttreatment versus the control DMSO treatment, immunostained for cytochrome *c*. (C and D) Quantitation of multinucleation in HeLa cells cosilenced with Myo19 and Mfn2 siRNAs versus the siControl RNA (mean of six experiments, keeping total amount of siRNA constant with siControl when necessary; 65 hr siRNA treatment time), with (D) showing the fragmented mitochondria caused by Mfn2 silencing in the experiment, stained as for (B). A similar fragmentation was seen in Mfn2 Myo19 RNAi cells (data not shown). (E and F) Quantitation of multinucleation in HeLa cells treated with 10 μM Mdivi with either Mfn2 or siControl silencing (mean of three experiments), with (F) showing representative cells, stained as for (B). Bars are SD in all graphs. Scale bars, 10 μm. Figure S2A shows a western blot showing reduced Mfn2 protein expression in the siMfn2-treated cells; Figures S2B–S2F illustrate the consequences of mitochondrial asymmetry for the subsequent cell cycle, showing that actomyosin ring structure and closure rates are not significantly affected by Myo19 depletion and that DNA bridges are not correlated with failure.

Given that Myo19 is localized to mitochondria, where it has been implicated in mitochondrial movement [4], it was important to test whether defects in the distribution or activity of mitochondria might be responsible for the observed Myo19 siRNA-induced disruption in cell division. Mitochondria are known to go through phases of fission and fusion during passage through the cell division cycle, which are thought to affect their activity and segregation [27, 28]. More specifically, mitochondrial fusion is thought to be accelerated at the G1-S transition, whereas fission is induced during mitosis [28]. Although we were unable to identify defects in cell division following drug-induced inhibition of oxidative phosphorylation (data not shown), we could phenocopy the multinucleation phenotype observed in Myo19 RNAi cells by treating HeLa cells with mitochondrial division inhibitor 1 (Mdivi), a drug that shifts the balance toward mitochondrial fusion through the inhibition of the fission machinery protein DRP1 [29] (Figure 2A, representative images in Figure 2B). This result implies that division failure could ultimately result from the simple obstruction of the actomyosin ring during cytokinesis by misplaced or excessively fused mitochondria in Myo19 RNAi cells. In support of this idea, both the Mdivi1 and the Myo19 depletion phenotypes were rescued in cells treated with siRNAs targeting mitofusin-2 (Mfn2), whose depletion shifts the balance toward mitochondrial fission [30] (Figures 2C and 2E, with a representative image in Figures 2D and 2F; see Figure S2A for evidence of Mfn2 protein reduction). A similar mitochondrial fragmentation was seen in Mfn2 Myo19 RNAi cells (data not shown). To determine the longer-term consequences of siMyo19-induced mitochondrial asymmetry, we followed dividing cell progeny to see how they coped with a subsequent cell cycle. HeLa cells stably expressing mitochondria-targeted yellow fluorescent protein (YFP) were treated with Myo19 siRNA or siControl and live imaged after 24 hr. As quantified in Figure S2B, Myo19 cells that failed division in the first round could produce daughter cells that succeeded in the second, and vice versa. Therefore, division failure appeared to be stochastic as expected if it was due to physical obstruction. To examine whether Myo19 is required for actomyosin ring formation or closure, we examined mitotic cells after depletion with siMyo19 or siControl siRNA and could see no obvious morphological differences in the contractile ring, as assessed by staining with an antibody specific for Anillin (Figure S2C). Moreover, using live imaging, we compared the time it took for cells to travel from the onset of anaphase to the onset of cytokinesis and observed no significant difference between control and Myo19 RNAi cells, whether or not the cell ultimately failed division (Figures S2D and S2E). To determine whether the cell division failure following Myo19 depletion might be due to improperly segregated chromosomes [31], we live imaged HeLa cells expressing histone-2B-mCherry after knockdown with siMyo19. Although we did observe lagging chromosomes in some control and Myo19 RNAi cells, there was no correlation between the presence of DNA bridges and division failure (Figure S2F). However, cells with more severe mitochondrial segregation defects tended to fail division at a higher rate (Figure S2F). Taken together with the mitochondrial fusion findings mentioned above, these results argue that Myo19 is not an integral part of the cell division machinery or of the actomyosin ring.

### Myo19 Ensures the Even Distribution and Segregation of Mitochondria

**Figure 3:**
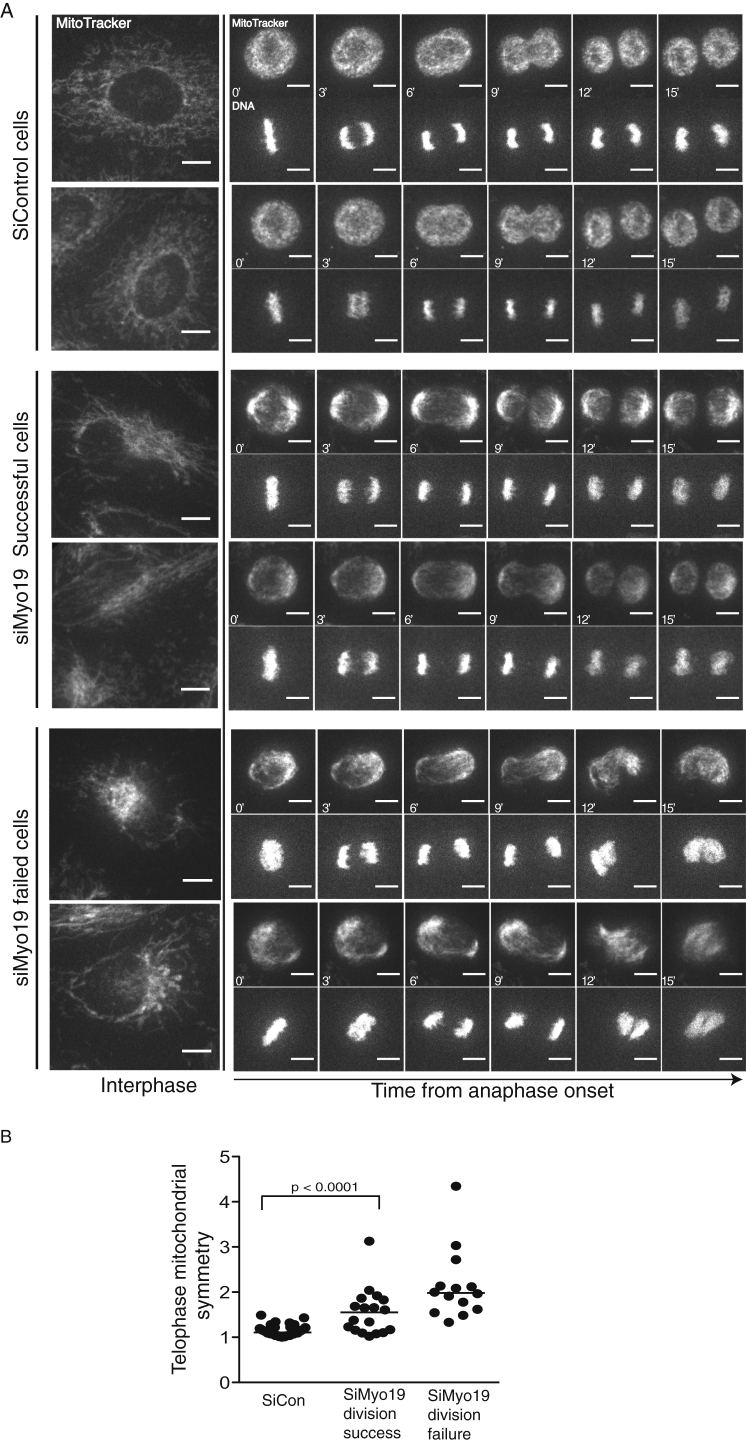
Myo19 Depletion Causes Mitochondria to Appear Clumped and to Be Asymmetrically Distributed in Both Metaphase and Anaphase (A) Time-lapse imaging (every 3 min at 2 μm sections) of HeLa cells stably expressing histone-2B-mCherry, treated with siCON (top set of cells) or siMyo19 (last two sets of cells) and labeled with MitoTracker Green 48 hr posttransfection. Images are maximum projections. Timestamps in minutes are indicated, with zero set during onset of early anaphase. Scale bars, 10 μm. (B) Quantitation of mitochondrial asymmetry in the same experiment at telophase in cells that succeed in division (center of graph) or at the time point prior to failure (right-hand side of graph). A number greater than 1 indicates relative asymmetry (see the Supplemental Experimental Procedures for details). See also Figure S3 for controls to show that the asymmetry in mitochondrial localization is independent of the state of fusion and can be rescued by the expression of murine Myo19 and Movies S1, S2, and S3.

During the course of these experiments, we noted an additional phenotype specific to Myo19 RNAi cells: mitochondria appeared asymmetrically localized in mitotic siMyo19 cells. Strikingly, while we observed relatively subtle changes in the structure and organization of mitochondria in interphase (Figure 3A, left panels), mitochondria appeared clumped and asymmetrically distributed in both metaphase and anaphase Myo19 RNAi cells (Figure 3A, montages; quantified in Figure 3B). As a result, pairs of daughter cells frequently inherited different proportions of the total mass of mitochondria (Figure S3A; this effect is quantified for anaphase in Figure S3B and quantified for telophase in Figure S3C; see also Movies S1, S2 and S3 for representative examples of an siControl cell, a Myo19 cell that succeeded in division, and a Myo19 cell that failed, respectively). This phenotype was statistically significant in experiments using a number of different Myo19-specific siRNAs (data not shown), was rescued by the expression of the RNAi-resistant BAC mouse GFP-fusion protein (Figure S3B, right side).

To determine whether the asymmetric segregation observed might be a simple consequence of defects in mitochondrial fission, we cosilenced Myo19 and Mfn2. While Mfn2 siRNA rescued the multinucleation phenotype seen in the Myo19 knockdown (Figure 2C), this increase in fission had no impact on the Myo19-RNAi-induced asymmetry in mitochondrial inheritance (Figure S3C, right side, with representative images as insets). This is important for two reasons. First, it strongly suggests that Myo19 does not exert its effects on mitochondrial segregation simply by altering the mitochondrial fusion status. Second, it enabled us to separate the effects of Myo19 on cell division, which appear to be due to a stochastic process in which misplaced mitochondria physical obstruct the process, from the robust defects in the symmetry of mitochondria at division. These distinct functions could be observed in movies of different dividing Myo19 RNAi HeLa cells stained with MitoTracker Green. As shown in Figure S3D, mitochondrial positioning was still visibly perturbed regardless whether division succeeds (middle row shows a representative cell) or fails (bottom row). Similar results were observed when live imaging was performed with a mitochondria-targeted YFP tag (HeLa-mito-YFP) instead of MitoTracker (data not shown).

**Figure 4:**
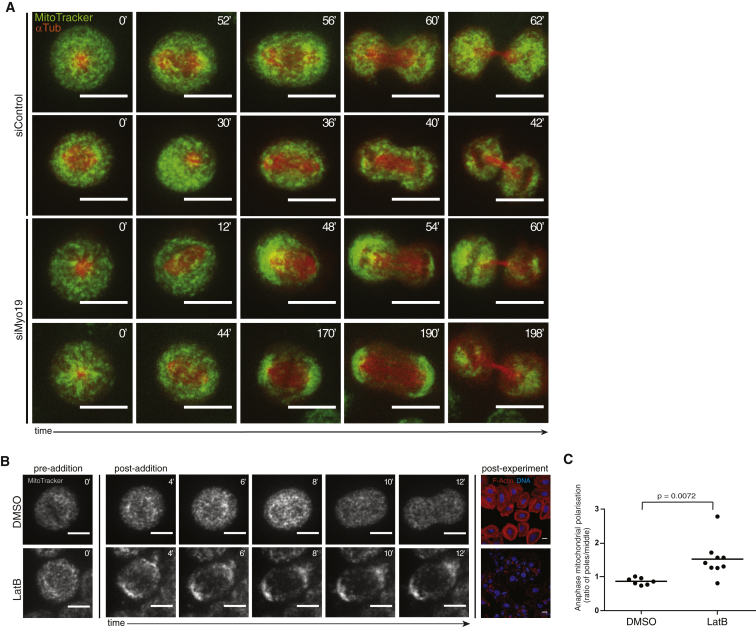
Myo19 Function Is Required during Mitosis Mitochondrial asymmetries arising during metaphase are corrected in control cells, but lead to the asymmetric segregation of mitochondria at division in Myo19-depleted cells. The disruption of filamentous actin leads to similar defects in mitochondrial movement at the onset of anaphase. (A) HeLa cells transiently expressing an mCherry-tagged α-tubulin protein (red) were synchronized in prometaphase with STLC, stained with MitoTracker Green, and followed after drug washout using high-resolution spinning disk confocal microscopy. Timestamps are in minutes. Row 1: a control cell with relatively symmetric mitochondrial distribution that stays symmetric through to cytokinesis. Row 2: symmetry is restored in a control cell with an initially asymmetric distribution of mitochondria prior to division. Rows 3 and 4: mitochondria tend to be relatively evenly distributed in prometaphase Myo19 RNAi cells but move rapidly to spindle poles just prior to the onset of anaphase, leading to a strikingly asymmetric segregation in some cases. Scale bars, 16 μm. (B) Mitochondrial movement in HeLa cells released from STLC treatment and live imaged with MitoTracker Green after treatment with the F-actin inhibitor latrunculin B or DMSO as a control; the label is MitoTracker Green in the grayscale images, before (left column, time zero) or after (middle column time series) addition of the indicated agent, every 2 min from 4 min postdrug treatment. The color images (right) show wells fixed at the end of the experiment (after 1.5 hr) and stained with DAPI for DNA (blue) and phalloidin for F-actin (red), to show that the latrunculin B completely dismantled F-actin in the treated cells. Scale bars, 10 μm. (C) Quantification of mitochondrial presence at the poles of anaphase HeLa cells in the experiment depicted in (B), compared with presence in the middle of the cell, expressed as a ratio. Movies were analyzed in “total sum” mode to capture pixels on all planes. See the Supplemental Experimental Procedures for quantification details. A number greater than 1 indicates polar enrichment. See also Figure S4 for quantification of mitochondrial presence at the poles of anaphase HeLa cells in cells live imaged after treatment with siMyo19 or siControl and subsequent STLC treatment and release, which show a similar latrunculin B phenocopy effect.

To determine whether Myo19 functions in mitosis to aid the proper distribution and segregation of mitochondria, we synchronized siMyo19 or siControl HeLa cells transiently expressing α-tubulin mCherry in prometaphase with the Eg5-inhibitor *S*-trityl-l-cysteine (STLC) [32] for 14 hr and then used MitoTracker Green to visualize mitochondria in the 30 min required for cells to exit mitosis following drug washout (Figure 4A; n = 15 for control and n = 15 for Myo19 RNAi). While control cells manifested occasional mild mitochondrial asymmetries at metaphase (e.g., second row for Figure 4A), they were able to restore symmetry during anaphase. In contrast, while the distribution of mitochondria appeared similar in the majority of control and siMyo19 cells arrested in prometaphase, following release from the arrest many Myo19 RNAi cells entering metaphase with a relatively normal mitochondrial distribution exhibited profound defects in mitochondrial segregation. These first arose as mitochondria moved to cell poles just prior to the onset of anaphase chromosomal movements (Figure 4A, bottom row) and led to defective mitochondrial segregation at division (Figure 4A, third row). In addition, it was noted that Myo19 cells tended to take longer on average to complete mitosis (data not shown). These data provide evidence that Myo19 functions to regulate mitochondrial movement and segregation as cells exit mitosis.

The correct partitioning of mitochondria into daughter cells during cell division requires precise orchestration, since defects in this process are likely to have serious consequences for daughter cells. Aside from their best-known role as producers of ATP, mitochondria regulate lipid and amino acid metabolism, redox regulation, calcium buffering, the production of heme, and apoptosis [33]. Given the fundamental and diverse nature of mitochondrial housekeeping functions, it is no surprise that their disruption is associated with numerous human diseases [3] and that checkpoints have been found in yeast that prevent division if problems in mitochondrial partitioning have occurred [34].

Eukaryotic cells ensure the fair partitioning of mitochondria using a variety of strategies. For example, in budding yeast, actin cables are employed to transport mitochondria from the mother cell to the bud cell [34], whereas in fission yeast, microtubules move the organelles to opposite poles [35]. The mechanisms in mammalian cells, however, remain poorly understood. RAL-induced fission of mitochondria in mitosis has very recently been proposed to contribute to their partitioning at cell division [36]. Although a recent paper [37] reported that mitochondria were recruited to the cleavage furrow during cytokinesis in HeLa and other cell types, a process its authors concluded was dependent on microtubules but not actin, we never observed this in our experiments (see, for example, Figures 3 and 4A; Figures S3A and S3D; Movie S1).

Given that Myo19 is an actin-binding protein [4], we wanted to test whether actin is required for the movement of mitochondria during early cell division. To this end, we synchronized HeLa cells transiently expressing α-tubulin mCherry in prometaphase with STLC for 14 hr and then stained with MitoTracker Green for the last 30 min. After drug washout, we imaged cells to inspect the behavior of mitochondria before and then 4 min after the addition of latrunculin B to disrupt F-actin assembly, or DMSO as a control (representative images in Figure 4B, with the quantification in Figure 4C). The disassembly of actin was found to trigger the rapid movement of mitochondria to the two opposing spindle poles of anaphase cells. Strikingly, this mirrored the premature movement of mitochondria to the spindle poles in Myo19 RNAi cells passing through mitosis (Figure 3A) and following the recovery from a prometaphase arrest (Figure 4A). This finding supports the idea that actin helps to control the movement of mitochondria during mitosis, but suggests that the actin cytoskeleton may constrain rather than promote mitochondrial movement in anaphase, which may be driven by microtubules [37].

Our data therefore support a mechanism whereby the mitochondrially associated myosin motor Myo19 functions together with F-actin in ensuring the regulated segregation of mitochondria during anaphase. This facilitates the equal inheritance of these organelles upon mitotic exit. It may also help to keep mitochondria out of the way of the cleavage plane to allow easy division of the cell body.

How might Myo19 move and position the mitochondria? In many other systems, general high-speed and longer-range transport of organelles on microtubules is refined by local anchorage to the actin cytoskeleton. For example, in the vertebrate axon, inhibition studies in vivo showed that mitochondrial transport can occur via either microtubules or actin microfilaments, but both are required for normal speed and net transport properties [38]. In this system, actin provides fine-tuning in response to signaling events; for example, nerve growth factor can induce the accumulation of the organelles in particular parts of the axon by recruiting mitochondria from the general microtubule-trafficked pool via filamentous actin tethers [39]. Similarly, melanosomes undergo kinesin-mediated radial transport from the center of the cell to its periphery. Once at the periphery, these organelles switch to Myo5-mediated transport and localization on actin filaments [40]. Such “track-switching” also occurs in the Myo5-mediated endoplasmic reticulum transport into dendrites underlying neuronal synaptic plasticity [41].

Our analysis supports a model whereby mitochondria move to the poles of the microtubule-based spindle at anaphase in a way that is independent of actin filaments, in line with the large body of literature showing that mitochondria are carried along microtubules in animal cells [42]. In this context, Myo19, through its association with mitochondria, may function to tether mitochondria to actin filaments during metaphase and anaphase, helping to regulate or limit their association with and poleward movement along microtubules, which provides the driving force for their segregation [27, 37]. This function would be similar to the role proposed for many other unconventional myosins as tethers [43].

Further experiments will be required to understand exactly how Myo19 regulates mitochondrial movement, and to ascertain whether its activity is coupled to mitotic progression—for example, to determine whether there is regulated disengagement of mitochondria from actin at anaphase. Taken together, however, our analysis shows that interactions between Myo19 and the actin cytoskeleton likely help to control the intracellular distribution of mitochondria and to ensure their precise and timely symmetrical segregation to spindle poles during animal cell division. Because mitochondria are unique among organelles in carrying their own genome, their missegregation, in common with errors in DNA segregation, would be expected to have profound effects on the future growth and viability of daughter cells [44].

## Author Contributions

J.L.R. contributed to the scientific strategy, performed experiments, helped analyze data, and wrote the manuscript. J.V.P. performed experiments and helped analyze the data. J.B. and B.N. provided expert technical assistance. N.M. and R.C.M. performed experiments. O.A.Q. performed the murine cell experiments, helped advise on the analysis of data, helped with the manuscript, and contributed to scientific strategy. J.E. advised and helped to oversee the RNAi screening. B.B. oversaw the scientific strategy and helped to design experiments, performed experiments, analyzed data, and cowrote the manuscript.

## Supplemental Information

Document S1. Supplemental Experimental Procedures, Figures S1–S4, and Table S1

Movie S1. A HeLa Cell Undergoing Normal Cell Division, Related to Figure 3 Time-lapse imaging (every 3 min in 2 μm sections) of HeLa cells stably expressing histone-2B-mCherry treated with control siRNA and stained with MitoTracker Green 48 hr posttransfection using high-resolution spinning disk confocal microscopy with a 60× objective. Note that the mitochondria are not clumped and that they are symmetrically segregated to both daughter cells.

Movie S2. A Myo19 RNAi Cell That Succeeds in Cell Division Despite the Clumped and Asymmetric Mitochondria, Related to Figure 3 Time-lapse imaging as for Movie S1 of HeLa cells treated with siMyo19 and labeled with MitoTracker Green (grayscale signal) 48 hr posttransfection. Note the dramatic switch in mitochondrial movement and positioning at the onset of anaphase, compared with the siControl movie.

Movie S3. A Myo19 RNAi Cell with Clumped and Asymmetric Mitochondria that Fails in Division, Related to Figure 3 Time-lapse imaging as for Movie S1 of HeLa cells treated with treated with siMyo19 and labeled with MitoTracker Green (green channel) 48 hr posttransfection and filmed after washout using high-resolution spinning disk confocal microscopy with the 60× objective. Note the asymmetric distribution of mitochondria.

Document S2. Article plus Supplemental Information
