# Supplementary material for: Myo19 Ensures Symmetric Partitioning of Mitochondria and Coupling of Mitochondrial Segregation to Cell Division
Source: Curr Biol. 2014 Nov 3;24(21):2598–605. doi: 10.1016/j.cub.2014.09.045 (PMC4228054; doi:10.1016/j.cub.2014.09.045)
Supplement: Document S1. Supplemental Experimental Procedures, Figures S1–S4, and Table S1 [file mmc1.pdf]

Current Biology, Volume *24*

Supplemental Information

An Actin-Based Myosin Motor, Myo19, Couples Mitochondrial Segregation to Cell Division

Jennifer L. Rohn, Jigna V. Patel, Beate Neumann, Jutta Bulkescher, Nunu Mchedlishvili, Rachel C. McMullan, Omar A. Quintero, Jan Ellenberg, and Buzz Baum

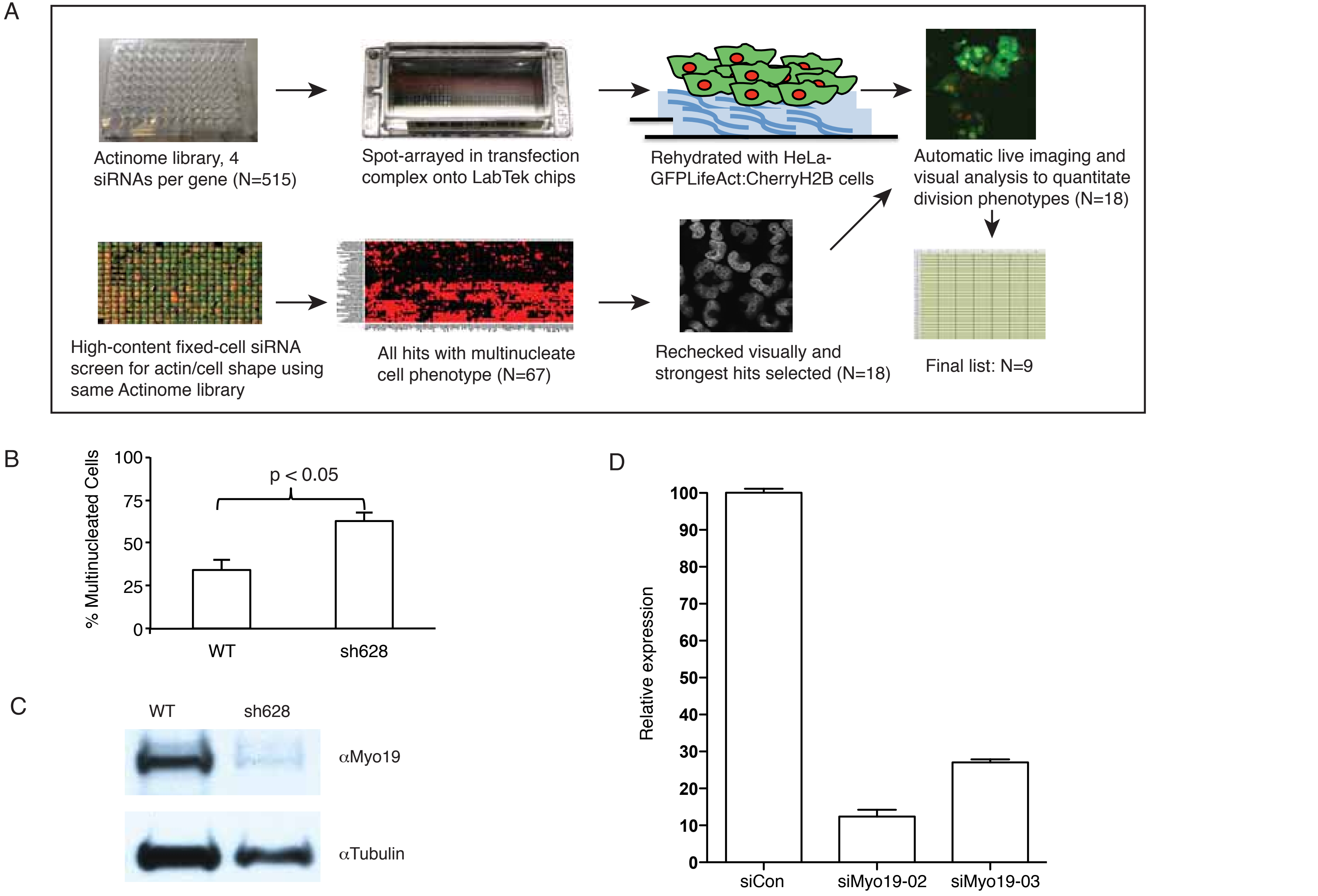

Supplemental Figure S1. Related to Figure 1. A live-imaging screen reveals genes important for normal cell division: strategy and validation. (A) Flowchart summarizing the live-image screen analysis. See Experimental Procedures and Results for details. (B) Stable expression of Myo19 shRNA in CAD cells leads to an increase in multinucleation ($p<0.05$, t-test, $n=5$, blind assay with at least 120 cells per replicate scored, mean with standard error shown). (C) Western blot confirms that shRNA expression in stable line sh628 CAD leads to a marked decrease in Myo19 protein; α-tubulin is probed in parallel as a loading control. (D) Quantitative PCR of HeLa cells silenced for Myo19 siRNAs compared with the siControl reagent. The graph shows relative expression units of amplimers produced by Myo19-specific primers after silencing by siRNA, normalized to actin amplimers. Experiment done three times, in triplicate; standard deviation from the mean is shown.

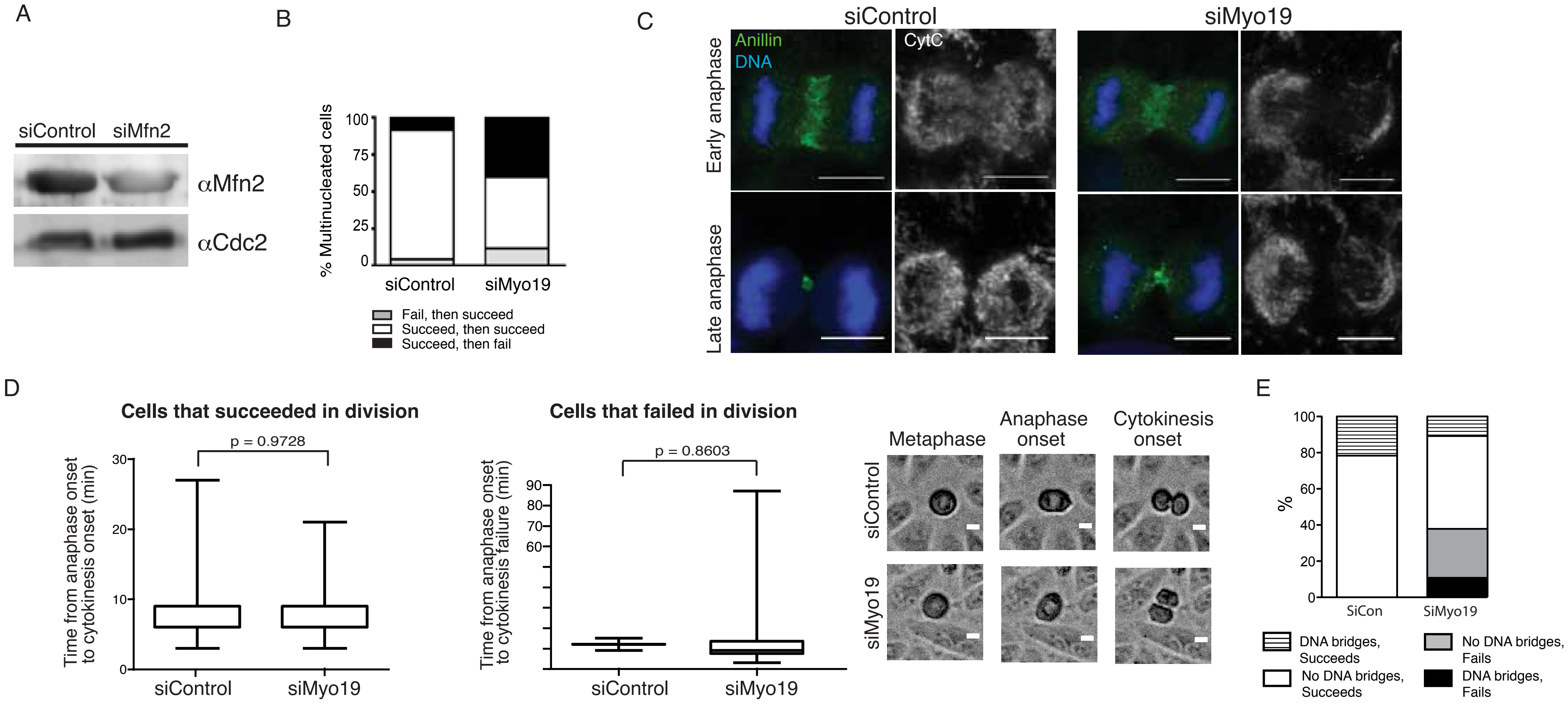

Supplemental Figure S2. Related to Figure 2. Myo19 depletion phenotype is rescued by promoting fission, does not perturb actomyosin ring structure or closure timing during anaphase, and is not influenced by DNA bridges. (A) Mfn2 siRNA treatment of HeLa cells leads to partial depletion of the Mfn2 protein. Western blot showing Mfn2 protein, compared with cdc2 housekeeping protein. (B) Myo19-induced mitochondrial asymmetry causes stochastic failures in cell division, so that cells that fail in one round can succeed in the subsequent round, and vice versa. HeLa-Mito-YFP cells were treated with siControl or siMyo19 and live-imaged 24 hours later every five minutes. Individual cells (n=23 for siControl n=42 for siMyo19) were followed through one mitosis and then their daughter cells, for a subsequent round of mitosis, to determine whether they succeeded or failed in division. (C) HeLa cells treated with Myo19 or siControl siRNA for 65 hours that ultimately succeeded in division were fixed and stained with DAPI for DNA (blue), antibody against Anillin (green), and against cytochrome C (grey); these reveal no obvious differences in the structure of the actomyosin ring before (top) or during (below) closure. (D) Cells that ultimately succeeded division following depletion for 48 hours with siMyo19 (n=163 for siMyo19 and n=162 for siControl RNAs), or alternatively failed division (n=37 for siMyo19 and n=2 for siControl) were scrutinized with live imaging and scored for how long they took from anaphase onset (defined as first deviation from spherical metaphase form, see images on right, central column) to cytokinesis onset (see images on right, right column. Images in first column show the frame before anaphase onset), or failure when relevant. Scalebar for images on right is 10 μm. The data shown are from the same experiments as in Figure 1B; please see details there. (E) HeLa cells stably expressing Histone-2B-mCherry, labelled with MitoTracker Green and treated with Myo19 (n=37) or siControl siRNA (n=57) as above were examined for the presence of DNA lagging or bridging during anaphase, and also scored for ultimate division outcome (success or fail).

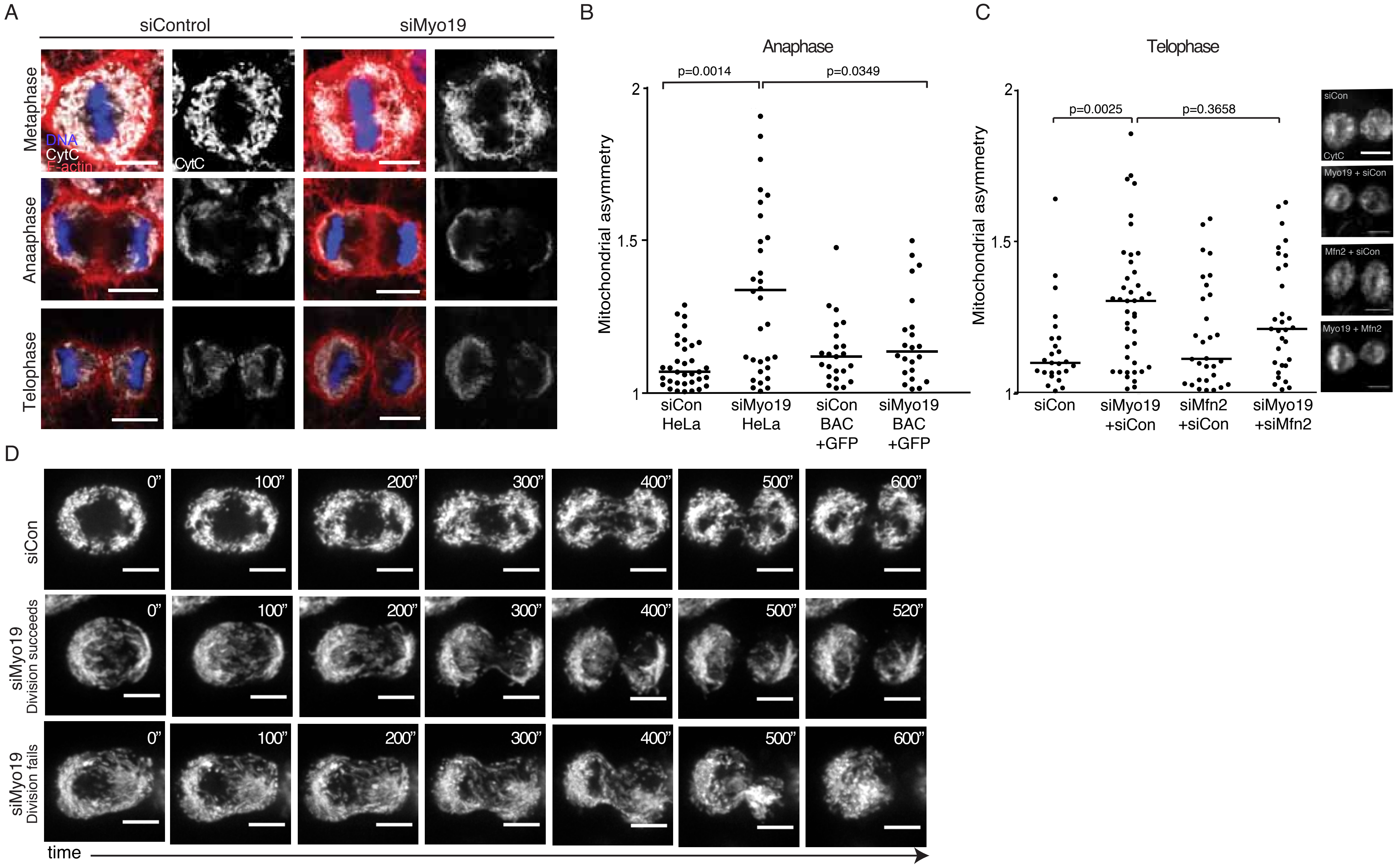

Supplementary Figure S3. Related to Figure 3. Myo19 depletion causes an asymmetric localization of mitochondria in multiple phases of cell division. (A) Cells treated with siControl or siMyo19 for 65 hr as indicated, at various stages of the cell cycle, fixed and stained with DAPI (blue), F-actin (red), and α-cytoch rome C (grey); greyscale counterparts are cytochrome C only. One plane through cell centre is shown. (B) Quantitation of mitochondrial asymmetry in anaphase of HeLa cells (left) or Myo19 BAC cells (right) treated with siCon or siMyo19 as indicated; the median is shown of at least 24 cells scored per condition. A number greater than 1 indicates relative asymme - try. (C) Analysis as for B but in HeLa telophase cells with the indicated siRNAs, scored in three different experiments. Insets to right show representative images (cytochrome C). (D) Timelapse imaging (every 10 sec) of HeLa cells treated with siRNA as indicated, labelled with MitoTracker Green 48 hr post-transfection. Images are maximum projections. Timestamps in seconds are indicated, with zero set during onset of anaphase. Scale bars are all 10 µm.

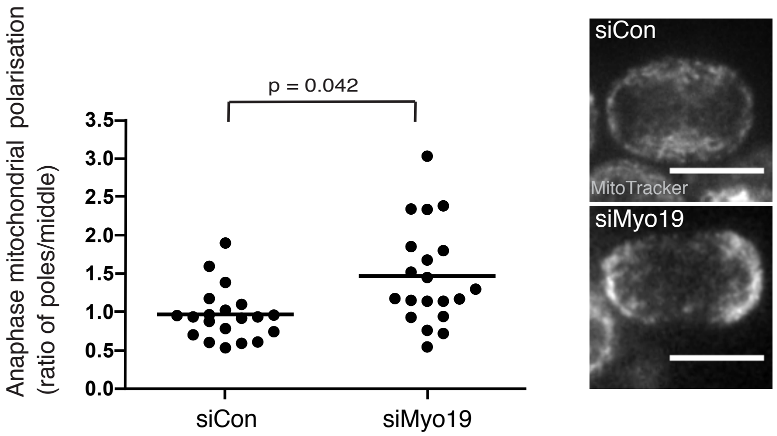

Supplementary Figure S4. Related to Figure 4. Lack of Myo19 results in mitochondrial accumulation at the spindle poles in anaphase. Quantification of mitochondrial presence at the poles of anaphase HeLa cells. This, like Figure 4A, phenocopies the effect of Latrunulin B treatment (as seen in Figure 4B). Movies were analysed in “total sum” mode to capture pixels in all planes, as in Figure 4C. A number greater than 1 indicates polar enrichment. On the right, representative images show Mitotracker Green labelling, Bar = 16 µm.

-

Gene Name	siRNA Name	Plate No.	Spot position	Replicates analyzed [d]	Sense Sequence	NCBI Accession No.	Catalogue No.	Mean % Defect[a,e]	StDev[f]
ANLN	ANLN-01	1	366	1,2	GGAGAUGGAUCAAGCAUUA	NM_018685	D-006838-01	85	9
ANLN	ANLN-02	1	79	NA [c]	GAAAUCCGCUUGCCUCUAA	NM_018685	D-006838-02		
ANLN	ANLN-03	1	175	NP [b]	GGAUAAAUCUGGCUAAUUG	NM_018685	D-006838-03		
ANLN	ANLN-05	1	270	1,3	ACGCAACACUUUUGAAUUA	NM_018685	D-006838-05	71	1
BCR	BCR-02	5	206	NA	GUAAAGCUCUCGGUCAAGU	NM_004327	D-003875-02	21	1
BCR	BCR-03	5	302	1,2	GCAUUCCGCUGACCAUCAA	NM_004327	D-003875-03	14	
BCR	BCR-06	5	218	1	CAGGAGCGCUUCCGCAUGA	NM_004327	D-003875-06	15	
BCR	BCR-13	5	314	1,2	CAGAAGAAGUGUUUCAGAA	NM_004327	D-003875-13		
CIT	CIT-01	1	244	1,3	GAUAUUAGAUGCCCUCUUU	NM_007174	D-004613-01	53	8
CIT	CIT-02	1	340	1,2	GGACCAGUCUUCAGUAUAA	NM_007174	D-004613-02	63	2
CIT	CIT-03	5	61	NA	GGACAUCUAUGCUAUGAAA	NM_007174	D-004613-03		
CIT	CIT-04	5	157	NA	GGAGCAGUCUUCCAAAUUU	NM_007174	D-004613-04		
ECT2	ECT2-01	6	195	1,3	GAUAAAGGAUGAUCUUGAA	NM_018098	D-006450-01	45	12
ECT2	ECT2-02	6	291	1,3	GCACUCACCUUGUAGUUGA	NM_018098	D-006450-02	68	26
ECT2	ECT2-03	6	207	NP	GAAGGGCUCUUAUGACAUC	NM_018098	D-006450-03		
ECT2	ECT2-04	6	303	NA	CAGAGGAGAUUAAGACUAU	NM_018098	D-006450-04		
MYO19	MYOHD1-01	3	226	NP	CGAUACAAGUUACUAAGAA	NM_025109	D-017137-01		
MYO19	MYOHD1-02	3	322	1,2,3	GGACCAAGGUGUUCAUGAC	NM_025109	D-017137-02	25	11
MYO19	MYOHD1-03	3	238	1,2,3	CAAUACGGCUAUGGGUGUA	NM_025109	D-017137-03	17	3
MYO19	MYOHD1-04	3	334	1,2,3	UCAGGGAUCGAUAAAGUUU	NM_025109	D-017137-04		
RACGAP1	RACGAP1-01	6	68	1,3	CAAAUUAUCUCUGAAGUGU	NM_013277	D-008650-01	41	13
RACGAP1	RACGAP1-02	6	164	NP	CCACAGACACCAGAUAUUA	NM_013277	D-008650-02		
RACGAP1	RACGAP1-03	6	260	1,3	GAACAUCAGCUUCUCAAGA	NM_013277	D-008650-03	27	7
RACGAP1	RACGAP1-04	6	356	1,3	GUAAUCAGGUGGAUGUAGA	NM_013277	D-008650-04	22	3
SPTBN1	SPTBN1-01	4	353	1,2,3	CGGAAGAGAUCGCCAAUUA	NM_003128	D-018149-01	25	16
SPTBN1	SPTBN1-02	4	65	NP	GACGAGAUCUUGUGGGUUG	NM_003128	D-018149-02		
SPTBN1	SPTBN1-03	4	161	NP	CUUAUGUGGUGACUUAUUA	NM_003128	D-018149-03		
SPTBN1	SPTBN1-04	4	257	1,2,3	CGAGUGCAAUGAAACCAAA	NM_003128	D-018149-04	14	5
SPTBN5	SPTBN5-01	4	271	NP	GCACAGACACCAGGACUUA	NM_016642	D-020903-01		
SPTBN5	SPTBN5-02	4	367	1	GGAAAGAUCUGGAGAGCGU	NM_016642	D-020903-02	16	
SPTBN5	SPTBN5-03	4	283	1,2,3	GCAGAAGUUUGGCCGAGAA	NM_016642	D-020903-03	20	7
SPTBN5	SPTBN5-04	4	379	1	CAACUGAGGCAGAGAUAUC	NM_016642	D-020903-04	15	
TPM4	TPM4-01	4	244	NP	CCAAGCACAUUGCGGAAGA	NM_003290	D-019753-01		
TPM4	TPM4-02	4	340	1,2,3	GCGGAGGUGUCUGAACUAA	NM_003290	D-019753-02	17	3
TPM4	TPM4-03	4	61	NP	GAACGUGGGCUUACAUCAG	NM_003290	D-019753-03	12	
TPM4	TPM4-04	4	157	1	GGAUCAGACACUAAACGAA	NM_003290	D-019753-04		

siCONTROL	OnTarget Plus non-targeting	1	131	1	Pool [g]	N/A	D-001810-01-05	6 [e]	1
siCONTROL	OnTarget Plus non-targeting	1	145	1	Pool [g]	N/A	D-001810-01-05		
siCONTROL	OnTarget Plus non-targeting	1	179	1	Pool [g]	N/A	D-001810-01-05		
siCONTROL	OnTarget Plus non-targeting	3	190	1	Pool [g]	N/A	D-001810-01-05	7	3
siCONTROL	OnTarget Plus non-targeting	3	179	1	Pool [g]	N/A	D-001810-01-05		
siCONTROL	OnTarget Plus non-targeting	3	145	1	Pool [g]	N/A	D-001810-01-05		
siCONTROL	OnTarget Plus non-targeting	4	145	1	Pool [g]	N/A	D-001810-01-05	9	3
siCONTROL	OnTarget Plus non-targeting	4	190	1	Pool [g]	N/A	D-001810-01-05		
siCONTROL	OnTarget Plus non-targeting	4	179	1	Pool [g]	N/A	D-001810-01-05		
siCONTROL	OnTarget Plus non-targeting	4	148	1	Pool [g]	N/A	D-001810-01-05		
siCONTROL	OnTarget Plus non-targeting	4	142	1	Pool [g]	N/A	D-001810-01-05		
siCONTROL	OnTarget Plus non-targeting	4	139	1	Pool [g]	N/A	D-001810-01-05		
siCONTROL	OnTarget Plus non-targeting	4	100	1	Pool [g]	N/A	D-001810-01-05		
siCONTROL	OnTarget Plus non-targeting	5	97	1	Pool [g]	N/A	D-001810-01-05	7	2
siCONTROL	OnTarget Plus non-targeting	5	142	1	Pool [g]	N/A	D-001810-01-05		
siCONTROL	OnTarget Plus non-targeting	5	179	1	Pool [g]	N/A	D-001810-01-05		
siCONTROL	OnTarget Plus non-targeting	6	100	1	Pool [g]	N/A	D-001810-01-05	13	5
siCONTROL	OnTarget Plus non-targeting	6	142	1	Pool [g]	N/A	D-001810-01-05		
siCONTROL	OnTarget Plus non-targeting	6	148	1	Pool [g]	N/A	D-001810-01-05		

[a] Based on starting multinucleation rates plus accumulated defects; see Experimental Procedures for details
[b] NP = No multinuclear phenotype in fixed screen
[c] NA = Not analyzed as phenotype very strong with known gene; results for two siRNAs deemed sufficient
[d] For genes, <3 replicates analyzed when it was not possible to score accurately due to quality control issues (focus drift or initial overcrowding)
[e] For siControl, different spots on same plate were analyzed, not replicates per spot
[f] No standard deviations are available in cases where only one film could be analyzed due to quality control issues
[g] The siCONTROL is a pool of four non-targeting sequences: UGGUUUACAUGUCGACUAA, UGGUUUACAUGUUGUGUGA, UGGUUUACAUGUUUUCUGA, and UGGUUUACAUGUUUUCCUA

Supplemental Table S1. Related to Figure 1. Details of individual siRNAs pooled in Figure 1, including their cell multinucleation phenotypes.

Supplemental Experimental Procedures

Cell culture. HeLa-Kyoto human carcinoma cells and HeLa-Kyoto cells stably expressing histome-2B-mCherry [S1] (a kind gift from M. Petronczki) were cultured at 37°C in a humidified incubator under 5% CO_2 in Dulbecco's Modified Eagle Medium (DMEM, Gibco 41965) supplemented with 10% fetal bovine serum (FBS) (PAA) and antibiotics (50 U/ml penicillin and 50 μg/ml streptomycin; Gibco 15070). HeLa cells stably expressing LifeAct-GFP/histone2B-mRFP (Hela-13) [S2] were grown the same except with the addition of puromycin (Sigma) to 0.5 μg/ml and G418 (Gibco) to 500 μg/ml to maintain selection of the transgenes. HeLa cells expressing mouse GFP-Myo19 (Myo19-BAC), a kind gift from A. Hyman, were cultured as above except the medium contained 400μg/ml G418. The murine neuronal tumor cell line CAD [S3] was cultured at 37°C in a humidified incubator under 5% CO2 in DMEM/F12 supplemented with 10% fetal bovine serum and antibiotics. shRNA stable cell lines were maintained in the same medium supplemented with 2μg/mL puromycin. To derive cells with red-fluorescent microtubules, we used FuGene HD (Promega) with a tubulin-mCherry plasmid. To establish HeLa cells stably expressing mitochondria-targeted TagYFP (HeLa-Mito-YFP), we transfected the Mito-TagYFP plasmid (a kind gift from Phong Tran) and derived single-cell clones using standard methods after selection in G418 (500μg/ml).

Antibody generation. Rabbit anti-mouse Myo19 antibodies were raised against amino acids 814-963 of the mouse Myo19 protein expressed in *E. coli* as a 6xHis fusion protein from the pQE30 vector. Purified protein was used as the immunogen and sent to Zymed (Invitrogen) for injection into rabbits. Anti-Myo19 antibodies were purified from rabbit serum using a Myo19 affinity resin generated from the same expressed protein.

siRNA library, live imaging screen and visual analysis. A custom Dharmacon siRNA library targeting 515 cytoskeletal genes and actin regulatory genes, four individual siRNAs per gene, was previously described, including the morphological phenotypes of their silencing in a fixed immunofluorescence assay [S4]. We arrayed siRNA spots using a VersArray chipwriter (Bio-Rad) in a “transfection-ready” format onto LabTek chamber slides in triplicate (384 spots per slide, total of 8 slides) exactly as previously described [S5]. Before live imaging, the grid was oriented with pen marks and seeded with HeLa13 cells. Two days after solid-phase transfection, each spot on the slide was imaged automatically on an Olympus automated epifluorescence microscope (IX-81; modified as described in [S5]) using the 10x objective, binning 1, every 33 minutes for 20 hours, using two channels (green; F-

actin and red; nucleus) per timepoint. We used the data from our previous fixed screen [S4] to focus on genes (N=67) whose knockdown had produced a multinucleated cell phenotype. We narrowed this list to the 18 most robust hits. Replicate films of these were analysed frame-by-frame and various aspects quantitated. First we scored multinucleation at the start of the film, to take into account phenotypes that were already well advanced by the start of filming. Then we scored the division outcome (multinucleation or normal) of at least 100 cells per film that contained a single nucleus at the start of the film and appeared otherwise normal. This cumulative multinucleation rate was added to the rate of multinucleation at the start of the film to generate a net percentage defect. The results for each independent siRNA targeting the same gene that scored positively in the screen were pooled to generate a mean multinucleation rate per gene (see Supplemental Table S1 for the phenotypes of individual siRNAs). Each slide of 384 spots contained at least three siControl siRNA spots, which were analysed and pooled. Using this approach, we identified candidates that exhibited division defects, with more than one independent siRNA, more frequently than was evident in cells treated with the siControl reagent. Depending on the siRNA, divisions failed at a variety of stages, from early anaphase all the way through to multinucleation resulting from daughter cell re-fusion during interphase.

Smaller-scale siRNA experiments. All siRNA experiments in HeLa and BAC cells were performed using reverse transfection with Lipfectamine-2000 (Life Technologies) according to the manufacturer's instructions, with Dharmcon siGenome duplexes (GE Healthcare). We tested the following siGenome siRNAs from the same company: Myo19-01, -02, -03, -04, -10, -11 and -12. Myo19-03 was used as the reagent once we had established that multiple siRNAs gave the same phenotypes (see the Results section for details; of this set, our trials indicated that only Myo19-01 and Myo19-12 had no phenotype). We also used an siRNA against mitofusion-2 (J-012961-07; sequence GCAACUCUAUCGUCACAGU). Our negative control was the OnTarget Plus control non-targeting siRNA Pool (Dharmacon/GE Healthcare, D-001810-01-05, referred to throughout the paper as siControl). We used the following conditions for transient transfection of HeLa and BAC cells: for 384-well black, thin-bottomed confocal-ready tissue-culture plates (Greiner bio-one): final volume of 60μL (final siRNA concentration of 25 nM) per well and 1000 cells; for 3 cm glass-bottomed dishes and 12-well glass-bottomed plates (Mattek): final volume of 300μL (final siRNA concentration of 20nM, containing 15,000 cells) per well, and topped up to 2mL after 6-8 hours. For transfections involving two siRNAs, the total amount of RNA was doubled and when compared with a single siRNA, the total amount was kept constant by the addition of an equal amount of siControl.

For experiments involving stable knockdown of Myo19, a MISSION plasmid for silencing the mouse Myo19 gene was purchased from Sigma (TRCN0000110628). To generate virus, HEK293FT cells were plated in T75 flasks at a concentration of approximately 5 million cells per plate and allowed to adhere overnight. Cells were then transfected with 1.25μg shRNA plasmid, 1.25μg PRRE plasmid, 1.25μg VSVG plasmid, 1.25μg REV plasmid (AddGene), and 0.2μg GFP-C1 plasmid (to verify transfection) using Fugene HD transfection reagent (Promega). After 24h the media in the flask was changed with 12ml fresh media. 24h later, the media was collected, and spun at 3000xg for 10 minutes. The supernatant was then filtered through a 0.45um filter and brought to 15ml. To infect CAD cells, a T25 flask that was ~70% confluent was treated with 3ml of virus-containing media supplemented with 8ug/ml hexamethadine bromide, and allowed to grow for two days. To select for stable incorporation of the plasmid DNA into the CAD cell genome, cells were then maintained in media containing 2μg/ml puromycin until a mixed population of cells with decreased Myo19 expression arose. Myo19 knockdown was verified via standard Western blotting using the polyclonal rabbit α-mouse Myo19 antibody at 0.17ug/ml overnight in TBS-0.1% Tween-20, or α-tubulin antibody (DM1A, Sigma) as a loading control, followed by the appropriate HRP-conjugated α-IgG antibodies and development using BioRad Clarity. Aside from its obvious strong and penetrant visual phenotype, Mfn2 depletion was assayed via standard Western blotting using a polyclonal rabbit antibody (H-68, sc-50331, Santa Cruz Biotechnology) diluted to 1:200; partial depletion was observed. The fact that virtually all mitochondria were fragmented after Mfn2 siRNA treatment suggests that expression of the family member Mfn1 cannot functionally compensate for even incomplete Mfn2 depletion in HeLa cells.

Quantitative PCR

Three days following siRNA treatments, we isolated RNA using QIAShredder and RNeasy (Qiagen) and prepared cDNA using a cDNA synthesis kit (Invitrogen), all according to the manufacturer's instructions. For quantitative PCR, we performed each reaction in triplicate using 150 ng DNA using the DyNAmo Flash SYBR Green qPCR kit (Qiagen). Primers used were: Myo19 Forward, 5' GGA CAA CCA GCC CTG TTT GGA TCT ; Myo19 Reverse, 5' TGG TCA GCT CAG GTG GGA TAG GGT; controls included standard primers for actin (a kind gift from Dan Cutler; Actin Forward 5' TGG TGG TGA AGC TGT AGCC 3'; Actin reverse 5' GCG AGA AGA TGA CCC AGAT 3') and reactions in which DNA was not included. Experiments using GAPDH as the standardization gene gave similar results (data not shown). Data were normalised by the delta-delta-CT method.

Cell staining and microscopy. For HeLa and BAC cell experiments, cells were fixed with freshly prepared formaldehyde (4% in PBS) for 20 mins at RT, permeablized with 0.2% Triton-X in PBS for 5 minutes and blocked with 5% BSA in PBS for 30 minutes. Depending on the experiment, we stained with TRITC-conjugated phalloidin (0.125μg/ml, Sigma P1951); DAPI (1 μg/ml, Sigma D9564), α-GFP (1:200; Invitrogen) and/or α-cytochrome C (1:200; Abcam) in a PBS solution containing 1% BSA for 1 hr. For antibodies, we used the appropriate fluorescently coupled Alexa-Fluor-α-IgG secondary antibodies from Molecular Probes at 1:500 for a further hour. After 3x washing with PBS, plates were stored in PBS containing 0.1% sodium azide (Sigma). Fixed images were acquired using a Leica SP5 or SPE3 scanning confocal microscope. All images were processed according to good practice using ImageJ/FIJI [S6].

For CAD experiments, cells were stained with 100nM Mitotracker Red CMXRos (Life Technologies) in DMEM for 15 minutes, followed by 2x 10-minute washes in DMEM and a quick rinse in PBS. Cells were then fixed in 4% paraformaldehyde (Electron Microscopy Sciences) in PBS for 10 minutes, permeabilized in PBS with 0.5% TritonX-100 (Sigma) for 5 minutes, and then stained with 75 nM DAPI and 7nM alexa-488-phalloidin (Molecular Probes) for 15 minutes. Coverslips were mounted in PBS containing 80% glycerol containing 0.5% N-propyl gallate. Cells were examined using a Olympus fluorescence microscope with a 60x/1.4NA and 40x/1.3NA objective with an attached Orca FLASH4v2 cooled CMOS digital camera (Hamamatsu) managed with Metamorph software, or a Nikon fluorescence microscope with a 60x/1.4NA objective with an attached Coolsnap HQ2 cooled CCD digital camera (Photometrics) managed by μManager [S7]. Images were analyzed using Metamorph or Fiji [S6].

Live imaging. For high-resolution live imaging of mitochondria, HeLa cells were transfected for 24 hours, after which MitoTracker Green (0.1 μM, Molecular Probes) was added for 30 mins before filming with the imaging medium (Fluorobrite DMEM [Gibco] + 10% FBS with the normal antibiotics as above). Cells were washed with PBS then imaged on an UltraVIEW VoX spinning disk confocal microscope (PerkinElmer) with the 60x objective lens every 20 sec (Supplemental Figure S3D) or every 2 min (Supplemental Figures S4 and Figure 4), or in the case of Supplemental Figure 4A, overnight filming with 3 min intervals and 2 μm sections. Cells for Figure 1B-C and Supplemental Figures S2B and D were prepared similarly except that imaging was conducted on an Axiovert 200M (Zeiss) with a motorized stage. For experiments involving synchronization at prometaphase, we used S-trityl-L-

cysteine (STLC; Sigma) at 5 μM treatment for 15 hr. When these experiments required additional manipulations, they were as follows: siRNAs were transfected 1 day before STLC addition, and plasmid transfected 12 hours before STLC addition. Next, MitoTracker Green was added for 30 mins, after which cells washed 5X with the imaging medium before filming. For the actin inhibition experiment after washing out the MitoTracker, we started filming, then added Latrunculin B (to 5 μM) or an equal volume of DMSO and resumed filming 14 minutes later at the same x/y point. Two hours later, we fixed and stained the cells as for HeLA and BACS, above, to confirm that the F-actin network had been completely dismantled by the Latrunculin treatment.

Quantitation of mitochondrial symmetry and spindle pole localization. In confocal images of dividing cells, Z-slices were summed for total pixel intensity in the channel corresponding to cytochrome C. Using ImageJ, two identical polygons were drawn to measure mean intensity, which was then multiplied by the area to get the total. Background intensity was subtracted, and the larger amount was divided by the smaller amount to obtain the mitochondrial ratio. A ratio of 1 would indicate symmetry, whereas higher amounts indicate asymmetry. To determine the ratio of mitochondria at spindle poles compared with the cell middle during anaphase, we used ImageJ to draw two identically sized polygons at each pole (cells viewed as "total sum"), to harvest all pixel information from all the stacks, and drew a third contiguous polygon in the middle that was exactly twice the size of each polar polygon. We measured mean intensity in all three polygons, multiplied this by each corresponding area to achieve the total, and then background intensity was subtracted. Then we added the pole numbers together and divided this number by the number for the middle polygon to obtain the spindle pole/middle ratio. A ratio greater than 1 would indicate an accumulation of mitochondria at the spindle poles.

Statistics. All statistics were performed using PRISM (GrafPad Software) or MiniCAD (Vectorworks).

Supplemental References

S1. Su, K.C., Takaki, T., and Petronczki, M. (2011). Targeting of the RhoGEF Ect2 to the equatorial membrane controls cleavage furrow formation during cytokinesis. Developmental cell *21*, 1104-1115.

S2. Matthews, H.K., Delabre, U., Rohn, J.L., Guck, J., Kunda, P., and Baum, B. (2012). Changes in Ect2 localization couple actomyosin-dependent cell shape changes to mitotic progression. Developmental cell *23*, 371-383.

S3. Qi, Y., Wang, J.K., McMillian, M., and Chikaraishi, D.M. (1997). Characterization of a CNS cell line, CAD, in which morphological differentiation is initiated by serum deprivation. J Neurosci *17*, 1217-1225.

S4. Rohn, J.L., Sims, D., Liu, T., Fedorova, M., Schock, F., Dopie, J., Vartiainen, M.K., Kiger, A.A., Perrimon, N., and Baum, B. (2011). Comparative RNAi screening identifies a conserved core metazoan actinome by phenotype. J Cell Biol *194*, 789-805.

S5. Neumann, B., Held, M., Liebel, U., Erfle, H., Rogers, P., Pepperkok, R., and Ellenberg, J. (2006). High-throughput RNAi screening by time-lapse imaging of live human cells. Nature methods *3*, 385-390.

S6. Schindelin, J., Arganda-Carreras, I., Frise, E., Kaynig, V., Longair, M., Pietzsch, T., Preibisch, S., Rueden, C., Saalfeld, S., Schmid, B., et al. (2012). Fiji: an open-source platform for biological-image analysis. Nature methods *9*, 676-682.

S7. Edelstein, A., Amodaj, N., Hoover, K., Vale, R., and Stuurman, N. (2010). Computer control of microscopes using microManager. Current protocols in molecular biology / edited by Frederick M. Ausubel ... [et al.] *Chapter 14*, Unit14 20.
