# Supplementary material for: Myo19 Ensures Symmetric Partitioning of Mitochondria and Coupling of Mitochondrial Segregation to Cell Division
Source: Curr Biol. 2014 Nov 3;24(21):2598–605. doi: 10.1016/j.cub.2014.09.045 (PMC4228054; doi:10.1016/j.cub.2014.09.045)
Supplement: Document S2. Article plus Supplemental Information [file mmc5.pdf]

Current Biology 24, 2598–2605, November 3, 2014 ©2014 The Authors http://dx.doi.org/10.1016/j.cub.2014.09.045

Report

# Myo19 Ensures Symmetric Partitioning of Mitochondria and Coupling of Mitochondrial Segregation to Cell Division

Jennifer L. Rohn,[1,7,*] Jigna V. Patel,[2,7] Beate Neumann,[3] Jutta Bulkescher,[4] Nunu Mchedlishvili,[2] Rachel C. McMullan,[5] Omar A. Quintero,[5] Jan Ellenberg,[6] and Buzz Baum[2,*]

[1]Centre for Clinical Science and Technology, Division of Medicine, University College London, Wolfson House, London NW1 2HE, UK
[2]MRC Laboratory for Molecular Cell Biology, University College London, London WC1E 6BT, UK
[3]Advanced Light Microscopy Facility, European Molecular Biology Laboratory (EMBL), Meyerhofstrasse 1, 69117 Heidelberg, Germany
[4]Novo Nordisk Foundation Center for Protein Research, Blegdamsvej 3B, 2200 Copenhagen, Denmark
[5]Department of Biology, University of Richmond, Richmond, VA 23173, USA
[6]Department of Cell Biology and Biophysics, European Molecular Biology Laboratory (EMBL), Meyerhofstrasse 1, 69117 Heidelberg, Germany

## Summary

**During animal cell division, an actin-based ring cleaves the cell into two. Problems with this process can cause chromosome missegregation and defects in cytoplasmic inheritance and the partitioning of organelles, which in turn are associated with human diseases [1–3]. Although much is known about how chromosome segregation is coupled to cell division, the way organelles coordinate their inheritance during partitioning to daughter cells is less well understood. Here, using a high-content live-imaging small interfering RNA screen, we identify Myosin-XIX (Myo19) as a novel regulator of cell division. Previously, this actin-based motor was shown to control the interphase movement of mitochondria [4]. Our analysis shows that Myo19 is indeed localized to mitochondria and that its silencing leads to defects in the distribution of mitochondria within cells and in mitochondrial partitioning at division. Furthermore, many Myo19 RNAi cells undergo stochastic division failure—a phenotype that can be mimicked using a treatment that blocks mitochondrial fission and rescued by decreasing mitochondrial fusion, implying that mitochondria can physically interfere with cytokinesis. Strikingly, using live imaging we also observe the inappropriate movement of mitochondria to the poles of spindles in cells depleted for Myo19 as they enter anaphase. Since this phenocopies the results of an acute loss of actin filaments in anaphase, these data support a model whereby the Myo19 actin-based motor helps to control mitochondrial movement to ensure their faithful segregation during division. The presence of DNA within mitochondria makes their inheritance an especially important aspect of symmetrical cell division.**

## Results and Discussion

To ensure faithful organelle inheritance, the segregation of each cellular component must be tightly coupled to the act of cell division. For chromosomes, this coupling relies on the exchange of signals between the elongating anaphase spindle and the overlying cell cortex, which helps to position the site at which the actomyosin-based ring is formed that cuts the cell into two [5]. Although the mechanisms are less well worked out, organelles may also rely on crosstalk between the microtubule-based spindle and the actin cortex for their partitioning [6–8]. To identify new actin-based regulators of cell division, we screened a human "actinome" small interfering RNA (siRNA) library [9] for siRNAs that induce division errors, targeting genes associated with the actin cytoskeleton, genes with predicted actin-binding domains, myosin motors, Rho family GTPases, GTPase activating proteins (GAPs), and guanine nucleotide exchange factors for siRNAs that induce division errors. While previous screens had used fixed endpoint assays to identify cytoskeletal regulators whose silencing led to cytokinesis failure (e.g., [10]), here we aimed to combine fixed data with live imaging to identify siRNAs that caused more subtle division errors.

Briefly, for the live-imaging analysis, a library targeting the human actinome, four siRNAs per gene, was mixed with a transfection reagent and arrayed in spots onto glass chamber slides [11]. HeLa-13 cells expressing LifeAct-EGFP to label filamentous actin and histone-2B-mCherry to label DNA [12] were then plated onto these arrays in triplicate experiments. Approximately 2 days after solid-phase reverse transfection, these marked islands of siRNA-treated cells were then filmed, using automated microscopy, to take a frame every 33 min over a 20 hr period. All images are freely available on our curated RNAi website FLIGHT.

We focused our manual screen analysis on hits (n = 67) that exhibited a multinucleated RNAi phenotype in the fixed screen carried out using the same library [9]. Movies were visually inspected to identify siRNAs inducing cell division defects. For the 18/67 hits with the most reproducible oligo-specific RNAi phenotypes, division outcome was scored for 100 cells in each film and was compared with the outcomes from siControl spots on the same slide. Using this approach, nine candidate genes were identified that exhibited a cell division defect with more than one independent siRNA (Figure 1A; for details of these siRNAs and their individual phenotypes, see Table S1 and see Figure S1A, available online, for a graphical depiction of the workflow).

The strongest hits corresponded to genes known to be crucial for faithful cell division, including Anillin [13], Citron kinase [14], and Ect2 [15]. The centralspindlin subunit Rac GTPase activating protein 1 (RACGAP1, MgcRacGAP) [16] was also identified as a moderately strong hit, together with two members of the beta-spectrin family [17], which bind actin and are major constituents of the cell cortex, and an unconventional myosin, Myosin-XIX (Myo19) [4]. Finally, BCR, which

[7]Co-first author
*Correspondence: j.rohn@ucl.ac.uk (J.L.R.), b.baum@ucl.ac.uk (B.B.)
This is an open access article under the CC BY license (http://creativecommons.org/licenses/by/3.0/).

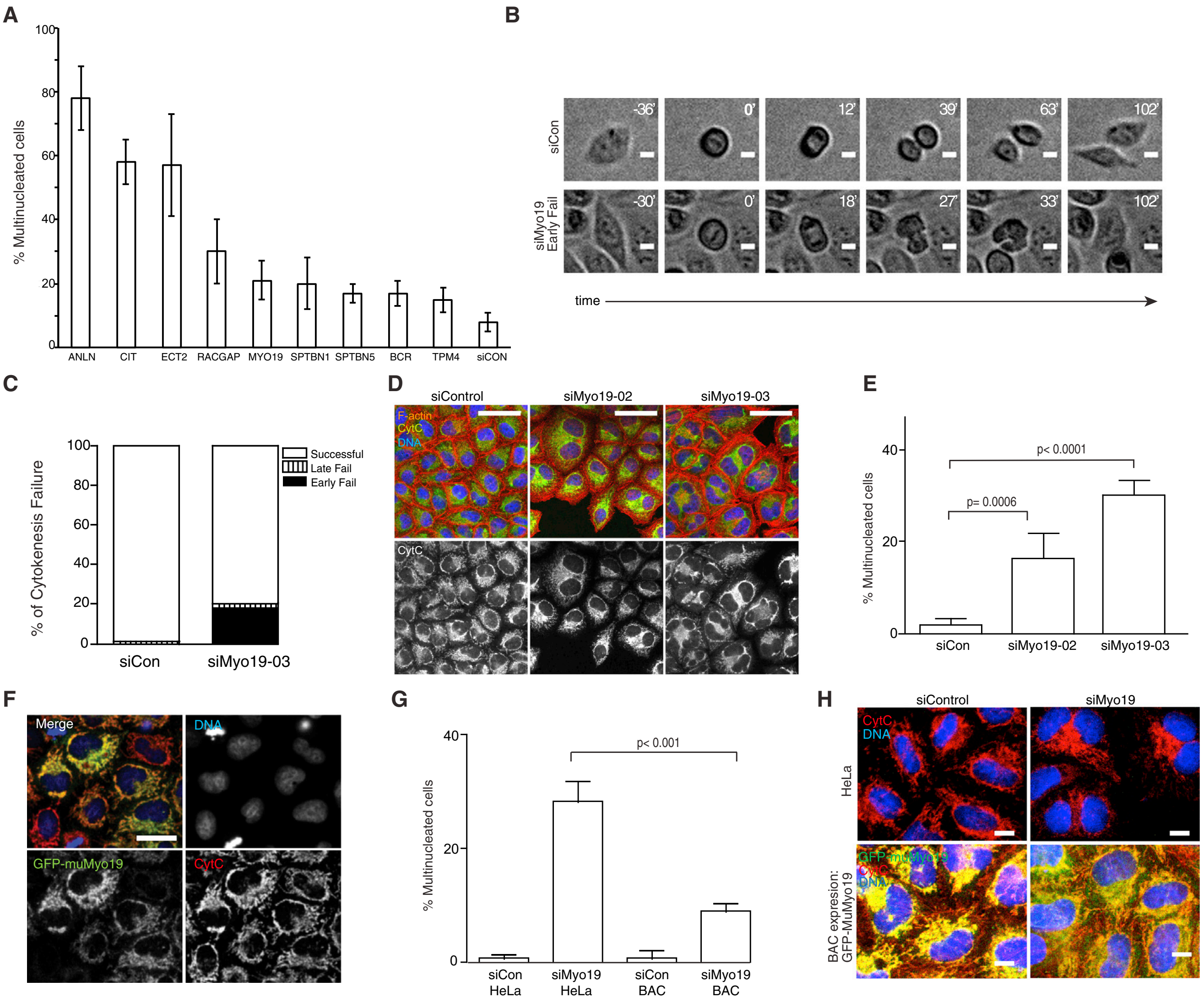


Figure 1. A Live-Image Screen Identifies a Role for Myo19 in Faithful Cell Division

(A) Division failure resulting in a multinucleated HeLa cell during live imaging from day 2 to day 3 postsilencing of various genes is shown relative to the negative siControl. The screen was performed in triplicate with four independent siRNAs per gene. The pooled mean of each gene is shown; bars indicate SD. See Figure S1A for a summary of the workflow and Table S1 for the individual siRNA phenotypes.

(B) Bright-field micrographs of cells live imaged every 3 min for 48 hr after silencing with the indicated siRNAs. Cell of interest is in middle of field; timestamp is in minutes, with zero set to the beginning of anaphase. Scale bars, 10 μm.

(C) Quantification of two experiments similar to that shown in (B) indicating mean percentage (at least 100 cells were counted per experiment) of cells that failed cell division early (soon after furrow formation) or late (later on in cytokinesis).

(D) Representative micrographs of HeLa cells silenced with the indicated siRNAs and fixed 65 hr posttransfection. DAPI staining (DNA) is blue, phalloidin (F-actin) is red, and cytochrome *c* is gray. Scale bars, 50 μm.

(E) Quantification of three experiments similar to (D) (at least 100 cells were counted per experiment; bars are SD).

(F) Micrographs of the HeLa BAC muMyo19 cell line, showing that the Myo19 division phenotype is rescued by a murine BAC copy of the gene. The grayscale images show individual channels as indicated; the color composite image shows DAPI staining (DNA; blue), cytochrome *c* (mitochondria; red), and GFP (muMyo19-GFP; green). Scale bar, 30 μm.

(G) Gene silencing of Myo19 compared with control in strongly GFP-positive HeLa BAC muMyo19 cells versus nontransgenic HeLa cells, showing that the muMyo19 can rescue the multinucleated cell phenotype caused by Myo19 knockdown alone. Experiment was performed three times with triplicate wells; the mean and SD of percent multinucleated cells is shown.

(H) Representative images from experiment performed in (G); same color channels as for (F). Scale bar, 10 μm. See Figures S1B and S1C for quantitation of the phenotype in an independent cell type and Figure S1D for quantitation of the siRNA silencing in HeLa cells by quantitative PCR.

See also Figure S1 and Table S1.

contains a C-terminal GAP domain specific for Rac [18], and TPM4 (tropomyosin 4 [19]) were recovered as relatively weak hits.

Given its potential novelty, we chose to focus our further analysis on the role of the unconventional myosin Myo19. Briefly, Myo19 is a myosin found in most animals [20] that appears to have been lost from lineages leading to insects and roundworms [21]. The 970 amino acid protein consists of a motor domain that has features distinguishing it from other myosin classes [22–24], a lever arm region containing three

light-chain-binding IQ-motifs, and a tail domain. Interestingly, the tail domain of Myo19, which is unique in the myosin family, was recently shown to target the protein to mitochondria [4]. This *Myo*19-specific *m*itochondria *o*uter *m*embrane *a*ssociation (MyMOMA) domain consists of approximately 150 amino acids (140 in mouse), bearing little easily identifiable sequence or structural homology to other known domains. Previous biochemical studies of purified Myo19 confirmed that the protein is a plus-end-directed [25], actin-activated ATPase capable of translocating actin filaments in vitro [22]. These studies also suggested that Myo19 may spend a large fraction of its chemomechanical cycle in an actin-bound state—a property common to transport motors such as Myo5 [26]. Moreover, the ectopic expression of full-length Myo19 was reported to increase the motility of mitochondria in interphase and to alter mitochondrial network organization in an actin-dependent fashion [4]. Given this, it was important to first confirm a role for Myo19 in division, before going on to test whether Myo19 might influence the outcome of cell division through its interaction with mitochondria.

## Myo19 Knockdown Leads to Cytokinesis Failure

Using a Myo19 siRNA (Myo19-03), we were able to confirm the increased rate of division failure following Myo19 silencing using live imaging (Figure 1B) and to show that in the majority of cases (37/41), division first failed soon after furrow formation, while only a small number of cells failed later (Figure 1C). To validate this Myo19 RNAi phenotype, we silenced the gene using nonoverlapping siRNAs. After fixation and staining, we observed a significant increase in the percentage of Myo19 RNAi cells with more than one nucleus compared with the nontargeting siControl (Figure 1D). Specifically, after 2 days of treatment with Myo19-02 siRNA, 14% of cells were multinucleated, whereas Myo19-03 siRNA caused 30% of the cells to contain multiple nuclei, compared to 2% of siControl-treated cells (Figure 1E). A similar phenotype was obtained using two additional siRNA sets targeting the same gene (data not shown).

To further validate the specificity of the Myo19 depletion phenotype, we performed a rescue experiment in a HeLa cell line stably expressing an N-terminally tagged GFP-bacterial artificial chromosome (BAC) version of murine Myo19, whose species-specific nucleotide sequence differences would make it resistant to knockdown by human Myo19 siRNA. In these cells, the entire visible pool of GFP-Myo19 was localized to mitochondria, as confirmed by colabeling with MitoTracker (data not shown) and by fixing and staining cells with an antibody against cytochrome *c* (Figure 1F). Moreover, in cells with robust mitochondrial GFP-Myo19 expression (Figure 1G, with representative images in Figure 1H), we observed a statistically significant reduction in the percentage of multinucleated cells following treatment with Myo19-03 siRNA, implying a direct or indirect role for this mitochondrially localized pool of Myo19 in cell division.

As additional validation, we observed a significant increase in the percentage of multinucleation in a different cell type, namely, murine cells expressing a stable, lentiviral-induced Myo19 small hairpin RNA (63% of silenced cells, compared with 34% in the control; Figure S1B), in which the Myo19 knockdown (of ~80%) could be verified by western blot analysis using an antibody generated against the mouse protein (Figure S1C). We also used quantitative PCR to validate the knockdown following treatment with Myo19-02 and Myo-19-03 siRNAs in HeLa cells, revealing an 88% reduction with Myo19-02 and a 73% reduction with Myo19-03 oligo relative to the siControl reagent (Figure S1D). From this point on, we used Myo19-03 as the representative siRNA for our experiments, as it had the most penetrant phenotype.

## Defects in Mitochondrial Organization Can Interfere with Cell Division

Given that Myo19 is localized to mitochondria, where it has been implicated in mitochondrial movement [4], it was important to test whether defects in the distribution or activity of mitochondria might be responsible for the observed Myo19 siRNA-induced disruption in cell division. Mitochondria are known to go through phases of fission and fusion during passage through the cell division cycle, which are thought to affect their activity and segregation [27, 28]. More specifically, mitochondrial fusion is thought to be accelerated at the G1-S transition, whereas fission is induced during mitosis [28]. Although we were unable to identify defects in cell division following drug-induced inhibition of oxidative phosphorylation (data not shown), we could phenocopy the multinucleation phenotype observed in Myo19 RNAi cells by treating HeLa cells with mitochondrial division inhibitor 1 (Mdivi), a drug that shifts the balance toward mitochondrial fusion through the inhibition of the fission machinery protein DRP1 [29] (Figure 2A, representative images in Figure 2B). This result implies that division failure could ultimately result from the simple obstruction of the actomyosin ring during cytokinesis by misplaced or excessively fused mitochondria in Myo19 RNAi cells. In support of this idea, both the Mdivi1 and the Myo19 depletion phenotypes were rescued in cells treated with siRNAs targeting mitofusin-2 (Mfn2), whose depletion shifts the balance toward mitochondrial fission [30] (Figures 2C and 2E, with a representative image in Figures 2D and 2F; see Figure S2A for evidence of Mfn2 protein reduction). A similar mitochondrial fragmentation was seen in Mfn2 Myo19 RNAi cells (data not shown). To determine the longer-term consequences of siMyo19-induced mitochondrial asymmetry, we followed dividing cell progeny to see how they coped with a subsequent cell cycle. HeLa cells stably expressing mitochondria-targeted yellow fluorescent protein (YFP) were treated with Myo19 siRNA or siControl and live imaged after 24 hr. As quantified in Figure S2B, Myo19 cells that failed division in the first round could produce daughter cells that succeeded in the second, and vice versa. Therefore, division failure appeared to be stochastic as expected if it was due to physical obstruction. To examine whether Myo19 is required for actomyosin ring formation or closure, we examined mitotic cells after depletion with siMyo19 or siControl siRNA and could see no obvious morphological differences in the contractile ring, as assessed by staining with an antibody specific for Anillin (Figure S2C). Moreover, using live imaging, we compared the time it took for cells to travel from the onset of anaphase to the onset of cytokinesis and observed no significant difference between control and Myo19 RNAi cells, whether or not the cell ultimately failed division (Figures S2D and S2E). To determine whether the cell division failure following Myo19 depletion might be due to improperly segregated chromosomes [31], we live imaged HeLa cells expressing histone-2B-mCherry after knockdown with siMyo19. Although we did observe lagging chromosomes in some control and Myo19 RNAi cells, there was no correlation between the presence of DNA bridges and division failure (Figure S2F). However, cells with more severe mitochondrial segregation defects tended to fail division at a higher rate (Figure S2F). Taken together with the mitochondrial fusion findings

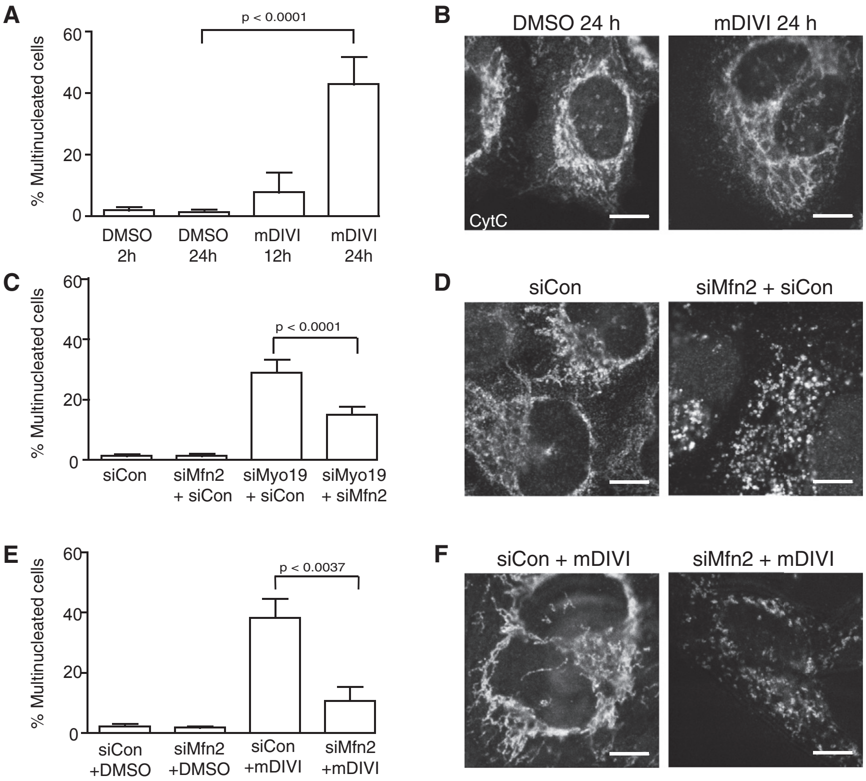


Figure 2. Inappropriate Mitochondrial Fusion Adversely Affects Cell Division, and Enhanced Fission Can Rescue the Myo19 Phenotype

(A and B) Quantification of multinucleation in HeLa cells treated with 10 μM Mdivi (100 cells were counted; mean of five experiments for 12 or 24 hr), with (B) showing representative cells at 24 hr posttreatment versus the control DMSO treatment, immunostained for cytochrome *c*.

(C and D) Quantitation of multinucleation in HeLa cells cosilenced with Myo19 and Mfn2 siRNAs versus the siControl RNA (mean of six experiments, keeping total amount of siRNA constant with siControl when necessary; 65 hr siRNA treatment time), with (D) showing the fragmented mitochondria caused by Mfn2 silencing in the experiment, stained as for (B). A similar fragmentation was seen in Mfn2 Myo19 RNAi cells (data not shown).

(E and F) Quantitation of multinucleation in HeLa cells treated with 10 μM Mdivi with either Mfn2 or siControl silencing (mean of three experiments), with (F) showing representative cells, stained as for (B). Bars are SD in all graphs. Scale bars, 10 μm. Figure S2A shows a western blot showing reduced Mfn2 protein expression in the siMfn2-treated cells; Figures S2B–S2F illustrate the consequences of mitochondrial asymmetry for the subsequent cell cycle, showing that actomyosin ring structure and closure rates are not significantly affected by Myo19 depletion and that DNA bridges are not correlated with failure.

mentioned above, these results argue that Myo19 is not an integral part of the cell division machinery or of the actomyosin ring.

## Myo19 Ensures the Even Distribution and Segregation of Mitochondria

During the course of these experiments, we noted an additional phenotype specific to Myo19 RNAi cells: mitochondria appeared asymmetrically localized in mitotic siMyo19 cells. Strikingly, while we observed relatively subtle changes in the structure and organization of mitochondria in interphase (Figure 3A, left panels), mitochondria appeared clumped and asymmetrically distributed in both metaphase and anaphase Myo19 RNAi cells (Figure 3A, montages; quantified in Figure 3B). As a result, pairs of daughter cells frequently inherited different proportions of the total mass of mitochondria (Figure S3A; this effect is quantified for anaphase in Figure S3B and quantified for telophase in FigureS3C; see also Movies S1, S2 and S3 for representative examples of an siControl cell, a Myo19 cell that succeeded in division, and a Myo19 cell that failed, respectively). This phenotype was statistically significant in experiments using a number of different Myo19-specific siRNAs (data not shown), was rescued by the expression of the RNAi-resistant BAC mouse GFP-fusion protein (Figure S3B, right side).

To determine whether the asymmetric segregation observed might be a simple consequence of defects in mitochondrial fission, we cosilenced Myo19 and Mfn2. While Mfn2 siRNA rescued the multinucleation phenotype seen in the Myo19 knockdown (Figure 2C), this increase in fission had no impact on the Myo19-RNAi-induced asymmetry in mitochondrial inheritance (Figure S3C, right side, with representative images as insets). This is important for two reasons. First, it strongly suggests that Myo19 does not exert its effects on mitochondrial segregation simply by altering the mitochondrial fusion status. Second, it enabled us to separate the effects of Myo19 on cell division, which appear to be due to a stochastic process in which misplaced mitochondria physical obstruct the process, from the robust defects in the symmetry of mitochondria at division. These distinct functions could be observed in movies of different dividing Myo19 RNAi HeLa cells stained with MitoTracker Green. As shown in Figure S3D, mitochondrial positioning was still visibly perturbed regardless whether division succeeds (middle row shows a representative cell) or fails (bottom row). Similar results were observed when live imaging was performed with a mitochondria-targeted YFP tag (HeLa-mito-YFP) instead of MitoTracker (data not shown).

To determine whether Myo19 functions in mitosis to aid the proper distribution and segregation of mitochondria, we synchronized siMyo19 or siControl HeLa cells transiently expressing α-tubulin mCherry in prometaphase with the Eg5-inhibitor *S*-trityl-L-cysteine (STLC) [32] for 14 hr and then used MitoTracker Green to visualize mitochondria in the 30 min required for cells to exit mitosis following drug washout (Figure 4A; n = 15 for control and n = 15 for Myo19 RNAi). While control cells manifested occasional mild mitochondrial asymmetries at metaphase (e.g., second row for Figure 4A), they were able to restore symmetry during anaphase. In contrast, while the distribution of mitochondria appeared similar in the majority of control and siMyo19 cells arrested in prometaphase, following release from the arrest many Myo19 RNAi cells entering metaphase with a relatively normal mitochondrial distribution exhibited profound defects in mitochondrial segregation. These first arose as mitochondria moved to cell poles just prior to the onset of anaphase chromosomal

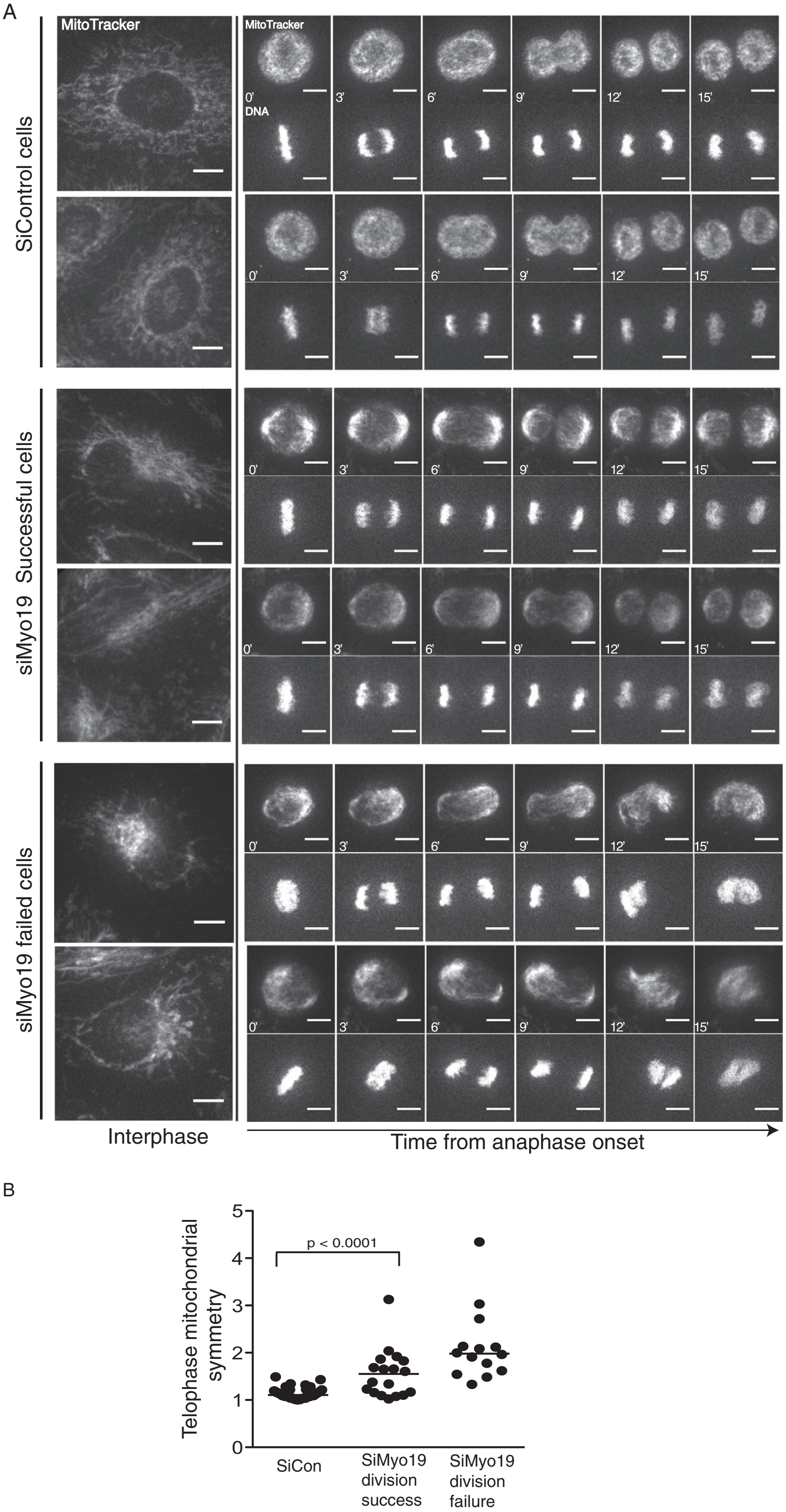


Figure 3. Myo19 Depletion Causes Mitochondria to Appear Clumped and to Be Asymmetrically Distributed in Both Metaphase and Anaphase

(A) Time-lapse imaging (every 3 min at 2 μm sections) of HeLa cells stably expressing histone-2B-mCherry, treated with siCON (top set of cells) or siMyo19 (last two sets of cells) and labeled with MitoTracker Green 48 hr posttransfection. Images are maximum projections. Timestamps in minutes are indicated, with zero set during onset of early anaphase. Scale bars, 10 μm.

(B) Quantitation of mitochondrial asymmetry in the same experiment at telophase in cells that succeed in division (center of graph) or at the time point prior to failure (right-hand side of graph). A number greater than 1 indicates relative asymmetry (see the Supplemental Experimental Procedures for details).

See also Figure S3 for controls to show that the asymmetry in mitochondrial localization is independent of the state of fusion and can be rescued by the expression of murine Myo19 and Movies S1, S2, and S3.

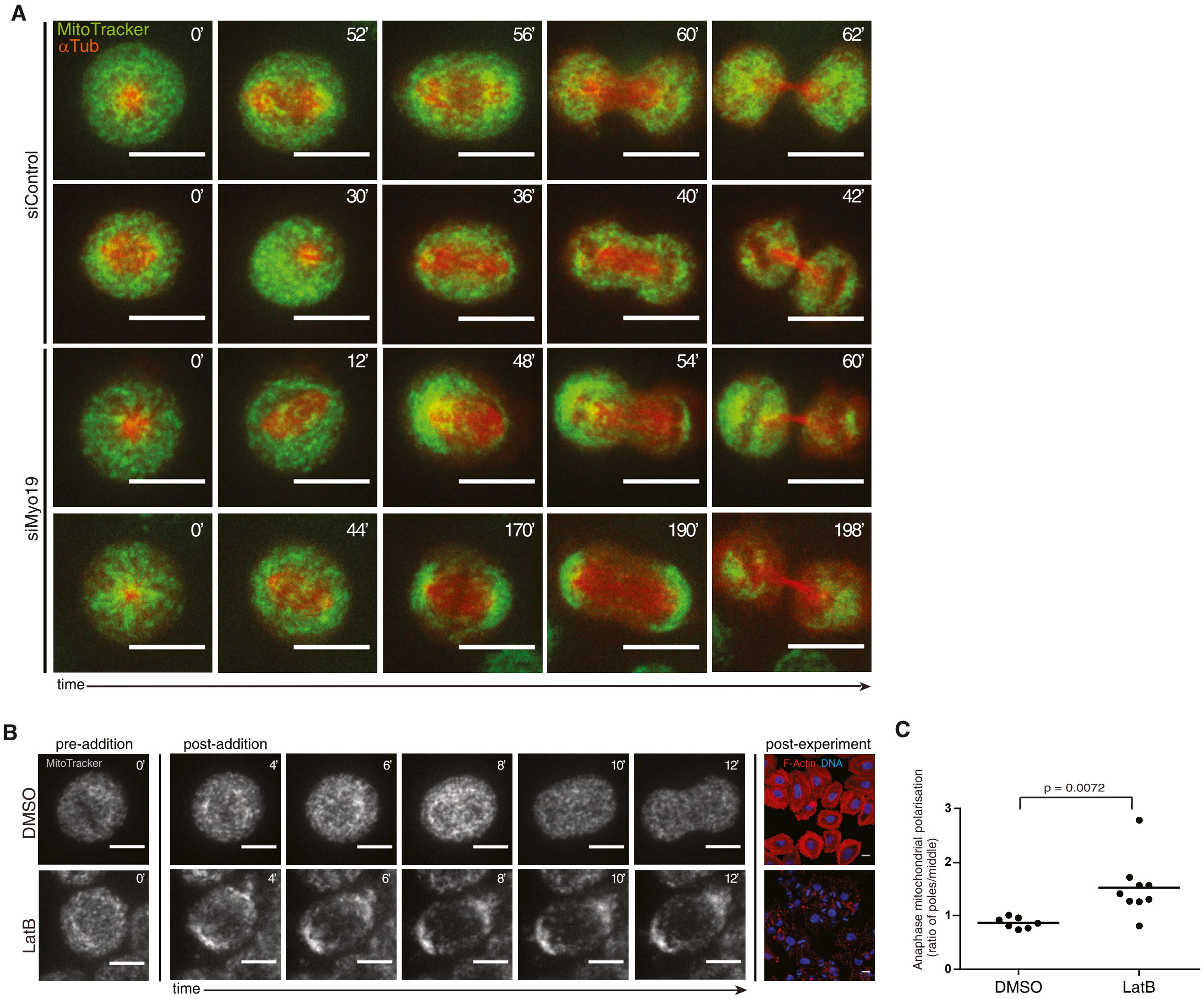


Figure 4. Myo19 Function Is Required during Mitosis

Mitochondrial asymmetries arising during metaphase are corrected in control cells, but lead to the asymmetric segregation of mitochondria at division in Myo19-depleted cells. The disruption of filamentous actin leads to similar defects in mitochondrial movement at the onset of anaphase.

(A) HeLa cells transiently expressing an mCherry-tagged α-tubulin protein (red) were synchronized in prometaphase with STLC, stained with MitoTracker Green, and followed after drug washout using high-resolution spinning disk confocal microscopy. Timestamps are in minutes. Row 1: a control cell with relatively symmetric mitochondrial distribution that stays symmetric through to cytokinesis. Row 2: symmetry is restored in a control cell with an initially asymmetric distribution of mitochondria prior to division. Rows 3 and 4: mitochondria tend to be relatively evenly distributed in prometaphase Myo19 RNAi cells but move rapidly to spindle poles just prior to the onset of anaphase, leading to a strikingly asymmetric segregation in some cases. Scale bars, 16 μm.

(B) Mitochondrial movement in HeLa cells released from STLC treatment and live imaged with MitoTracker Green after treatment with the F-actin inhibitor latrunculin B or DMSO as a control; the label is MitoTracker Green in the grayscale images, before (left column, time zero) or after (middle column time series) addition of the indicated agent, every 2 min from 4 min postdrug treatment. The color images (right) show wells fixed at the end of the experiment (after 1.5 hr) and stained with DAPI for DNA (blue) and phalloidin for F-actin (red), to show that the latrunculin B completely dismantled F-actin in the treated cells. Scale bars, 10 μm.

(C) Quantification of mitochondrial presence at the poles of anaphase HeLa cells in the experiment depicted in (B), compared with presence in the middle of the cell, expressed as a ratio. Movies were analyzed in “total sum” mode to capture pixels on all planes. See the Supplemental Experimental Procedures for quantification details. A number greater than 1 indicates polar enrichment.

See also Figure S4 for quantification of mitochondrial presence at the poles of anaphase HeLa cells in cells live imaged after treatment with siMyo19 or siControl and subsequent STLC treatment and release, which show a similar latrunculin B phenocopy effect.

movements (Figure 4A, bottom row) and led to defective mitochondrial segregation at division (Figure 4A, third row). In addition, it was noted that Myo19 cells tended to take longer on average to complete mitosis (data not shown). These data provide evidence that Myo19 functions to regulate mitochondrial movement and segregation as cells exit mitosis.

The correct partitioning of mitochondria into daughter cells during cell division requires precise orchestration, since defects in this process are likely to have serious consequences for daughter cells. Aside from their best-known role as producers of ATP, mitochondria regulate lipid and amino acid metabolism, redox regulation, calcium buffering, the

production of heme, and apoptosis [33]. Given the fundamental and diverse nature of mitochondrial housekeeping functions, it is no surprise that their disruption is associated with numerous human diseases [3] and that checkpoints have been found in yeast that prevent division if problems in mitochondrial partitioning have occurred [34].

Eukaryotic cells ensure the fair partitioning of mitochondria using a variety of strategies. For example, in budding yeast, actin cables are employed to transport mitochondria from the mother cell to the bud cell [34], whereas in fission yeast, microtubules move the organelles to opposite poles [35]. The mechanisms in mammalian cells, however, remain poorly understood. RAL-induced fission of mitochondria in mitosis has very recently been proposed to contribute to their partitioning at cell division [36]. Although a recent paper [37] reported that mitochondria were recruited to the cleavage furrow during cytokinesis in HeLa and other cell types, a process its authors concluded was dependent on microtubules but not actin, we never observed this in our experiments (see, for example, Figures 3 and 4A; Figures S3A and S3D; Movie S1).

Given that Myo19 is an actin-binding protein [4], we wanted to test whether actin is required for the movement of mitochondria during early cell division. To this end, we synchronized HeLa cells transiently expressing $\alpha$-tubulin mCherry in prometaphase with STLC for 14 hr and then stained with MitoTracker Green for the last 30 min. After drug washout, we imaged cells to inspect the behavior of mitochondria before and then 4 min after the addition of latrunculin B to disrupt F-actin assembly, or DMSO as a control (representative images in Figure 4B, with the quantification in Figure 4C). The disassembly of actin was found to trigger the rapid movement of mitochondria to the two opposing spindle poles of anaphase cells. Strikingly, this mirrored the premature movement of mitochondria to the spindle poles in Myo19 RNAi cells passing through mitosis (Figure 3A) and following the recovery from a prometaphase arrest (Figure 4A). This finding supports the idea that actin helps to control the movement of mitochondria during mitosis, but suggests that the actin cytoskeleton may constrain rather than promote mitochondrial movement in anaphase, which may be driven by microtubules [37].

Our data therefore support a mechanism whereby the mitochondrially associated myosin motor Myo19 functions together with F-actin in ensuring the regulated segregation of mitochondria during anaphase. This facilitates the equal inheritance of these organelles upon mitotic exit. It may also help to keep mitochondria out of the way of the cleavage plane to allow easy division of the cell body.

How might Myo19 move and position the mitochondria? In many other systems, general high-speed and longer-range transport of organelles on microtubules is refined by local anchorage to the actin cytoskeleton. For example, in the vertebrate axon, inhibition studies in vivo showed that mitochondrial transport can occur via either microtubules or actin microfilaments, but both are required for normal speed and net transport properties [38]. In this system, actin provides fine-tuning in response to signaling events; for example, nerve growth factor can induce the accumulation of the organelles in particular parts of the axon by recruiting mitochondria from the general microtubule-trafficked pool via filamentous actin tethers [39]. Similarly, melanosomes undergo kinesin-mediated radial transport from the center of the cell to its periphery. Once at the periphery, these organelles switch to Myo5-mediated transport and localization on actin filaments [40]. Such "track-switching" also occurs in the Myo5-mediated endoplasmic reticulum transport into dendrites underlying neuronal synaptic plasticity [41].

Our analysis supports a model whereby mitochondria move to the poles of the microtubule-based spindle at anaphase in a way that is independent of actin filaments, in line with the large body of literature showing that mitochondria are carried along microtubules in animal cells [42]. In this context, Myo19, through its association with mitochondria, may function to tether mitochondria to actin filaments during metaphase and anaphase, helping to regulate or limit their association with and poleward movement along microtubules, which provides the driving force for their segregation [27, 37]. This function would be similar to the role proposed for many other unconventional myosins as tethers [43].

Further experiments will be required to understand exactly how Myo19 regulates mitochondrial movement, and to ascertain whether its activity is coupled to mitotic progression—for example, to determine whether there is regulated disengagement of mitochondria from actin at anaphase. Taken together, however, our analysis shows that interactions between Myo19 and the actin cytoskeleton likely help to control the intracellular distribution of mitochondria and to ensure their precise and timely symmetrical segregation to spindle poles during animal cell division. Because mitochondria are unique among organelles in carrying their own genome, their missegregation, in common with errors in DNA segregation, would be expected to have profound effects on the future growth and viability of daughter cells [44].

### Supplemental Information

Supplemental Information includes Supplemental Experimental Procedures, four figures, one table, and three movies and can be found with this article online at http://dx.doi.org/10.1016/j.cub.2014.09.045.

### Author Contributions

J.L.R. contributed to the scientific strategy, performed experiments, helped analyze data, and wrote the manuscript. J.V.P. performed experiments and helped analyze the data. J.B. and B.N. provided expert technical assistance. N.M. and R.C.M. performed experiments. O.A.Q. performed the murine cell experiments, helped advise on the analysis of data, helped with the manuscript, and contributed to scientific strategy. J.E. advised and helped to oversee the RNAi screening. B.B. oversaw the scientific strategy and helped to design experiments, performed experiments, analyzed data, and cowrote the manuscript.

### Acknowledgments

J.L.R. thanks the Wellcome Trust for financial support. J.V.P. and B.B. were supported by Cancer Research UK. O.Q. thanks the National Cancer Institute (K01CA160667) and the University of Richmond. We are grateful to Kristine Schauer, Sacha Sarfati, Juanma Garcia, Clotilde Cadart, Phong Tran and his team, and members of the B.B. lab for helpful discussions; Ina Poser and Tony Hyman for providing the Myo19-BAC cell line; Mark Petronczki for providing HeLa Kyoto H2B-mCherry cells; Dan Cutler's lab for providing actin primers; and Phong Tran for providing the Mito-Tag-YFP plasmid. We also thank Jos de Graaf and Christian Conrad for technical assistance in the live-cell screening process.

Received: April 4, 2014
Revised: July 24, 2014
Accepted: September 12, 2014
Published: October 23, 2014

### References

1. Duijf, P.H., and Benezra, R. (2013). The cancer biology of whole-chromosome instability. Oncogene *32*, 4727–4736.

2. Nunnari, J., and Suomalainen, A. (2012). Mitochondria: in sickness and in health. Cell *148*, 1145–1159.
3. Raimundo, N. (2014). Mitochondrial pathology: stress signals from the energy factory. Trends Mol. Med. *20*, 282–292.
4. Quintero, O.A., DiVito, M.M., Adikes, R.C., Kortan, M.B., Case, L.B., Lier, A.J., Panaretos, N.S., Slater, S.Q., Rengarajan, M., Feliu, M., and Cheney, R.E. (2009). Human Myo19 is a novel myosin that associates with mitochondria. Curr. Biol. *19*, 2008–2013.
5. Barr, F.A., and Gruneberg, U. (2007). Cytokinesis: placing and making the final cut. Cell *131*, 847–860.
6. Poteryaev, D., Squirrell, J.M., Campbell, J.M., White, J.G., and Spang, A. (2005). Involvement of the actin cytoskeleton and homotypic membrane fusion in ER dynamics in Caenorhabditis elegans. Mol. Biol. Cell *16*, 2139–2153.
7. Lowe, M., and Barr, F.A. (2007). Inheritance and biogenesis of organelles in the secretory pathway. Nat. Rev. Mol. Cell Biol. *8*, 429–439.
8. Fagarasanu, A., and Rachubinski, R.A. (2007). Orchestrating organelle inheritance in Saccharomyces cerevisiae. Curr. Opin. Microbiol. *10*, 528–538.
9. Rohn, J.L., Sims, D., Liu, T., Fedorova, M., Schöck, F., Dopie, J., Vartiainen, M.K., Kiger, A.A., Perrimon, N., and Baum, B. (2011). Comparative RNAi screening identifies a conserved core metazoan actinome by phenotype. J. Cell Biol. *194*, 789–805.
10. Zhang, X., Bedigian, A.V., Wang, W., and Eggert, U.S. (2012). G protein-coupled receptors participate in cytokinesis. Cytoskeleton (Hoboken) *69*, 810–818.
11. Neumann, B., Held, M., Liebel, U., Erfle, H., Rogers, P., Pepperkok, R., and Ellenberg, J. (2006). High-throughput RNAi screening by time-lapse imaging of live human cells. Nat. Methods *3*, 385–390.
12. Matthews, H.K., Delabre, U., Rohn, J.L., Guck, J., Kunda, P., and Baum, B. (2012). Changes in Ect2 localization couple actomyosin-dependent cell shape changes to mitotic progression. Dev. Cell *23*, 371–383.
13. Piekny, A.J., and Maddox, A.S. (2010). The myriad roles of Anillin during cytokinesis. Semin. Cell Dev. Biol. *21*, 881–891.
14. Matsumura, F. (2005). Regulation of myosin II during cytokinesis in higher eukaryotes. Trends Cell Biol. *15*, 371–377.
15. Tatsumoto, T., Xie, X., Blumenthal, R., Okamoto, I., and Miki, T. (1999). Human ECT2 is an exchange factor for Rho GTPases, phosphorylated in G2/M phases, and involved in cytokinesis. J. Cell Biol. *147*, 921–928.
16. Lekomtsev, S., Su, K.C., Pye, V.E., Blight, K., Sundaramoorthy, S., Takaki, T., Collinson, L.M., Cherepanov, P., Divecha, N., and Petronczki, M. (2012). Centralspindlin links the mitotic spindle to the plasma membrane during cytokinesis. Nature *492*, 276–279.
17. Baines, A.J. (2009). Evolution of spectrin function in cytoskeletal and membrane networks. Biochem. Soc. Trans. *37*, 796–803.
18. Narayanan, A.S., Reyes, S.B., Um, K., McCarty, J.H., and Tolias, K.F. (2013). The Rac-GAP Bcr is a novel regulator of the Par complex that controls cell polarity. Mol. Biol. Cell *24*, 3857–3868.
19. Lin, J.J., Eppinga, R.D., Warren, K.S., and McCrae, K.R. (2008). Human tropomyosin isoforms in the regulation of cytoskeleton functions. Adv. Exp. Med. Biol. *644*, 201–222.
20. Sebé-Pedrós, A., Grau-Bové, X., Richards, T.A., and Ruiz-Trillo, I. (2014). Evolution and classification of myosins, a paneukaryotic whole-genome approach. Genome Biol. Evol. *6*, 290–305.
21. Odronitz, F., and Kollmar, M. (2007). Drawing the tree of eukaryotic life based on the analysis of 2,269 manually annotated myosins from 328 species. Genome Biol. *8*, R196.
22. Adikes, R.C., Unrath, W.C., Yengo, C.M., and Quintero, O.A. (2013). Biochemical and bioinformatic analysis of the myosin-XIX motor domain. Cytoskeleton (Hoboken) *70*, 281–295.
23. Cope, M.J., Whisstock, J., Rayment, I., and Kendrick-Jones, J. (1996). Conservation within the myosin motor domain: implications for structure and function. Structure *4*, 969–987.
24. Berg, J.S., Powell, B.C., and Cheney, R.E. (2001). A millennial myosin census. Mol. Biol. Cell *12*, 780–794.
25. Lu, Z., Ma, X.N., Zhang, H.M., Ji, H.H., Ding, H., Zhang, J., Luo, D., Sun, Y., and Li, X.D. (2014). Mouse myosin-19 is a plus-end-directed, high-duty ratio molecular motor. J. Biol. Chem. *289*, 18535–18548.
26. Howard, J. (1997). Molecular motors: structural adaptations to cellular functions. Nature *389*, 561–567.
27. Detmer, S.A., and Chan, D.C. (2007). Functions and dysfunctions of mitochondrial dynamics. Nat. Rev. Mol. Cell Biol. *8*, 870–879.
28. Mitra, K., Wunder, C., Roysam, B., Lin, G., and Lippincott-Schwartz, J. (2009). A hyperfused mitochondrial state achieved at G1-S regulates cyclin E buildup and entry into S phase. Proc. Natl. Acad. Sci. USA *106*, 11960–11965.
29. Cassidy-Stone, A., Chipuk, J.E., Ingerman, E., Song, C., Yoo, C., Kuwana, T., Kurth, M.J., Shaw, J.T., Hinshaw, J.E., Green, D.R., and Nunnari, J. (2008). Chemical inhibition of the mitochondrial division dynamin reveals its role in Bax/Bak-dependent mitochondrial outer membrane permeabilization. Dev. Cell *14*, 193–204.
30. Zorzano, A., Liesa, M., Sebastián, D., Segalés, J., and Palacín, M. (2010). Mitochondrial fusion proteins: dual regulators of morphology and metabolism. Semin. Cell Dev. Biol. *21*, 566–574.
31. Norden, C., Mendoza, M., Dobbelaere, J., Kotwaliwale, C.V., Biggins, S., and Barral, Y. (2006). The NoCut pathway links completion of cytokinesis to spindle midzone function to prevent chromosome breakage. Cell *125*, 85–98.
32. Skoufias, D.A., DeBonis, S., Saoudi, Y., Lebeau, L., Crevel, I., Cross, R., Wade, R.H., Hackney, D., and Kozielski, F. (2006). S-trityl-L-cysteine is a reversible, tight binding inhibitor of the human kinesin Eg5 that specifically blocks mitotic progression. J. Biol. Chem. *281*, 17559–17569.
33. McBride, H.M., Neuspiel, M., and Wasiak, S. (2006). Mitochondria: more than just a powerhouse. Curr. Biol. *16*, R551–R560.
34. Peraza-Reyes, L., Crider, D.G., and Pon, L.A. (2010). Mitochondrial manoeuvres: latest insights and hypotheses on mitochondrial partitioning during mitosis in Saccharomyces cerevisiae. BioEssays *32*, 1040–1049.
35. Yaffe, M.P., Stuurman, N., and Vale, R.D. (2003). Mitochondrial positioning in fission yeast is driven by association with dynamic microtubules and mitotic spindle poles. Proc. Natl. Acad. Sci. USA *100*, 11424–11428.
36. Kashatus, D.F., Lim, K.H., Brady, D.C., Pershing, N.L., Cox, A.D., and Counter, C.M. (2011). RALA and RALBP1 regulate mitochondrial fission at mitosis. Nat. Cell Biol. *13*, 1108–1115.
37. Lawrence, E.J., and Mandato, C.A. (2013). Mitochondria localize to the cleavage furrow in mammalian cytokinesis. PLoS ONE *8*, e72886.
38. Morris, R.L., and Hollenbeck, P.J. (1995). Axonal transport of mitochondria along microtubules and F-actin in living vertebrate neurons. J. Cell Biol. *131*, 1315–1326.
39. Chada, S.R., and Hollenbeck, P.J. (2004). Nerve growth factor signaling regulates motility and docking of axonal mitochondria. Curr. Biol. *14*, 1272–1276.
40. Hume, A.N., and Seabra, M.C. (2011). Melanosomes on the move: a model to understand organelle dynamics. Biochem. Soc. Trans. *39*, 1191–1196.
41. Hammer, J.A., 3rd, and Sellers, J.R. (2012). Walking to work: roles for class V myosins as cargo transporters. Nat. Rev. Mol. Cell Biol. *13*, 13–26.
42. Saxton, W.M., and Hollenbeck, P.J. (2012). The axonal transport of mitochondria. J. Cell Sci. *125*, 2095–2104.
43. Hartman, M.A., Finan, D., Sivaramakrishnan, S., and Spudich, J.A. (2011). Principles of unconventional myosin function and targeting. Annu. Rev. Cell Dev. Biol. *27*, 133–155.
44. Johnston, I.G., Gaal, B., Neves, R.P., Enver, T., Iborra, F.J., and Jones, N.S. (2012). Mitochondrial variability as a source of extrinsic cellular noise. PLoS Comput. Biol. *8*, e1002416.

**Current Biology, Volume *24***
**Supplemental Information**

# An Actin-Based Myosin Motor, Myo19, Couples Mitochondrial Segregation to Cell Division

**Jennifer L. Rohn, Jigna V. Patel, Beate Neumann, Jutta Bulkescher, Nunu Mchedlishvili, Rachel C. McMullan, Omar A. Quintero, Jan Ellenberg, and Buzz Baum**

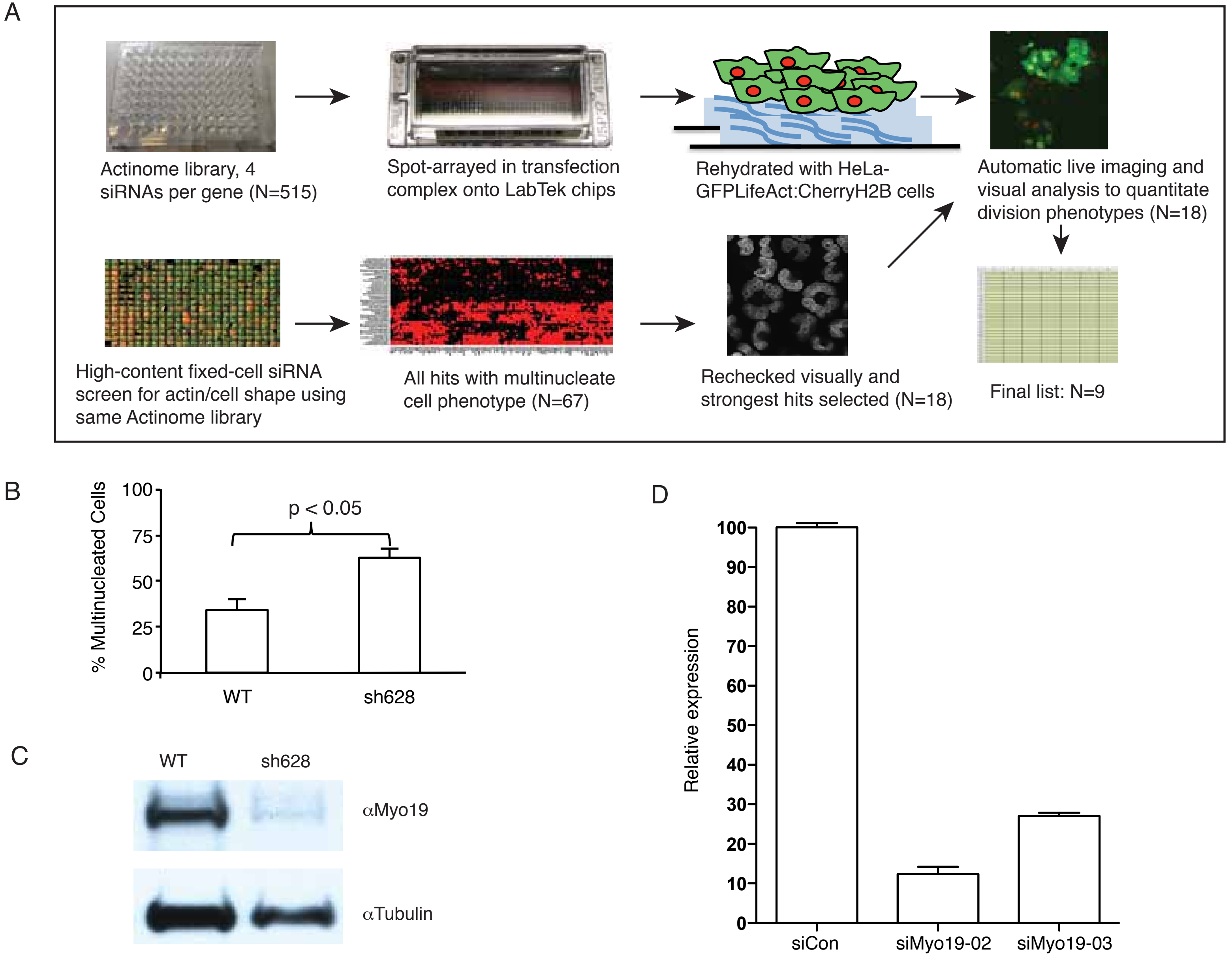


**Supplemental Figure S1**. Related to Figure 1. A live-imaging screen reveals genes important for normal cell division: strategy and validation. (A) Flowchart summarizing the live-image screen analysis. See Experimental Procedures and Results for details. (B) Stable expression of Myo19 shRNA in CAD cells leads to an increase in multinucleation ($p<0.05$, t-test, $n=5$, blind assay with at least 120 cells per replicate scored, mean with standard error shown). (C) Western blot confirms that shRNA expression in stable line sh628 CAD leads to a marked decrease in Myo19 protein; α-tubulin is probed in parallel as a loading control. (D) Quantitative PCR of HeLa cells silenced for Myo19 siRNAs compared with the siControl reagent. The graph shows relative expression units of amplimers produced by Myo19-specific primers after silencing by siRNA, normalized to actin amplimers. Experiment done three times, in triplicate; standard deviation from the mean is shown.

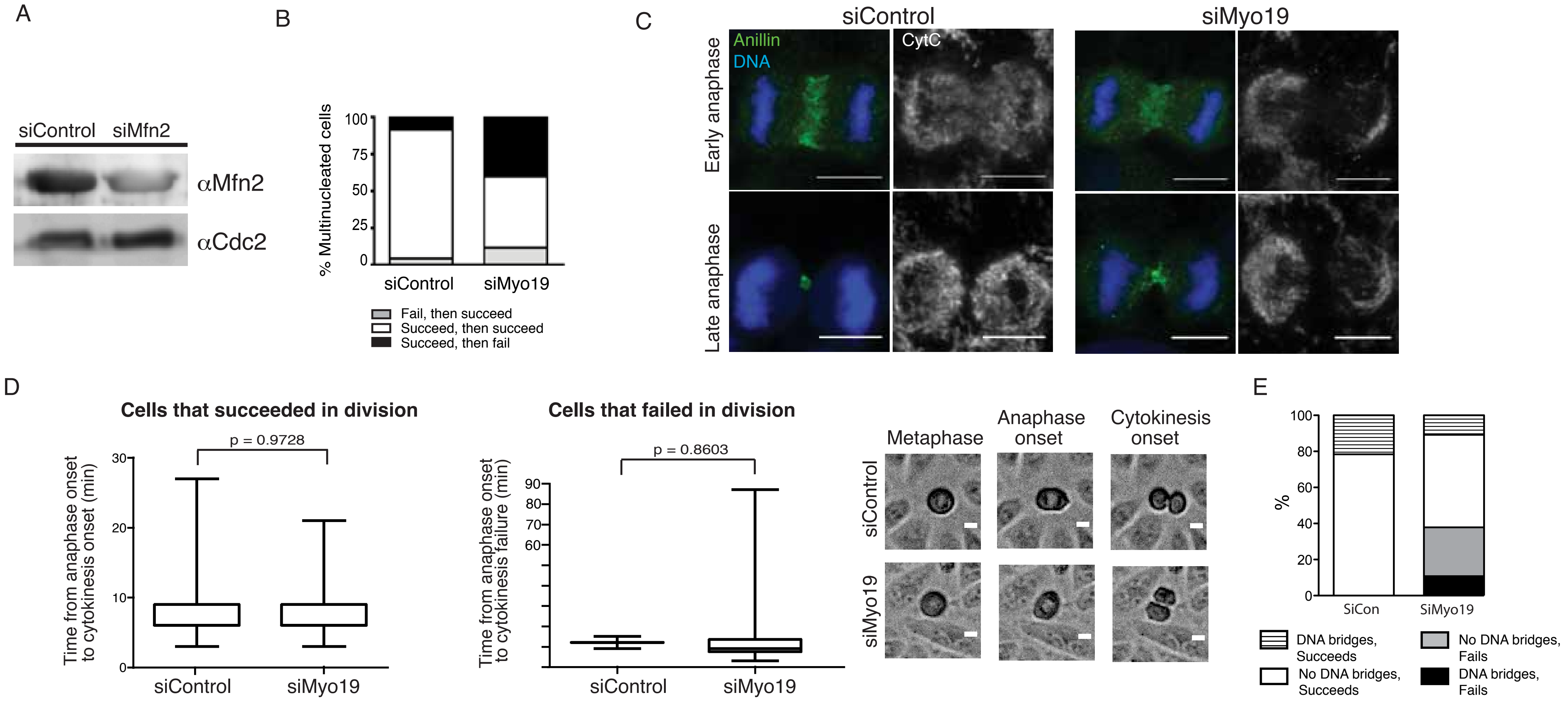


**Supplemental Figure S2.** Related to Figure 2. Myo19 depletion phenotype is rescued by promoting fission, does not perturb actomyosin ring structure or closure timing during anaphase, and is not influenced by DNA bridges. (A) Mfn2 siRNA treatment of HeLa cells leads to partial depletion of the Mfn2 protein. Western blot showing Mfn2 protein, compared with cdc2 housekeeping protein. (B) Myo19-induced mitochondrial asymmetry causes stochastic failures in cell division, so that cells that fail in one round can succeed in the subsequent round, and vice versa. HeLa-Mito-YFP cells were treated with siControl or siMyo19 and live-imaged 24 hours later every five minutes. Individual cells (n=23 for siControl n=42 for siMyo19) were followed through one mitosis and then their daughter cells, for a subsequent round of mitosis, to determine whether they succeeded or failed in division. (C) HeLa cells treated with Myo19 or siControl siRNA for 65 hours that ultimately succeeded in division were fixed and stained with DAPI for DNA (blue), antibody against Anillin (green), and against cytochrome C (grey); these reveal no obvious differences in the structure of the actomyosin ring before (top) or during (below) closure. (D) Cells that ultimately succeeded division following depletion for 48 hours with siMyo19 (n=163 for siMyo19 and n=162 for siControl RNAs), or alternatively failed division (n=37 for siMyo19 and n=2 for siControl) were scrutinized with live imaging and scored for how long they took from anaphase onset (defined as first deviation from spherical metaphase form, see images on right, central column) to cytokinesis onset (see images on right, right column. Images in first column show the frame before anaphase onset), or failure when relevant. Scalebar for images on right is 10 μm. The data shown are from the same experiments as in Figure 1B; please see details there. (E) HeLa cells stably expressing Histone-2B-mCherry, labelled with MitoTracker Green and treated with Myo19 (n=37) or siControl siRNA (n=57) as above were examined for the presence of DNA lagging or bridging during anaphase, and also scored for ultimate division outcome (success or fail).

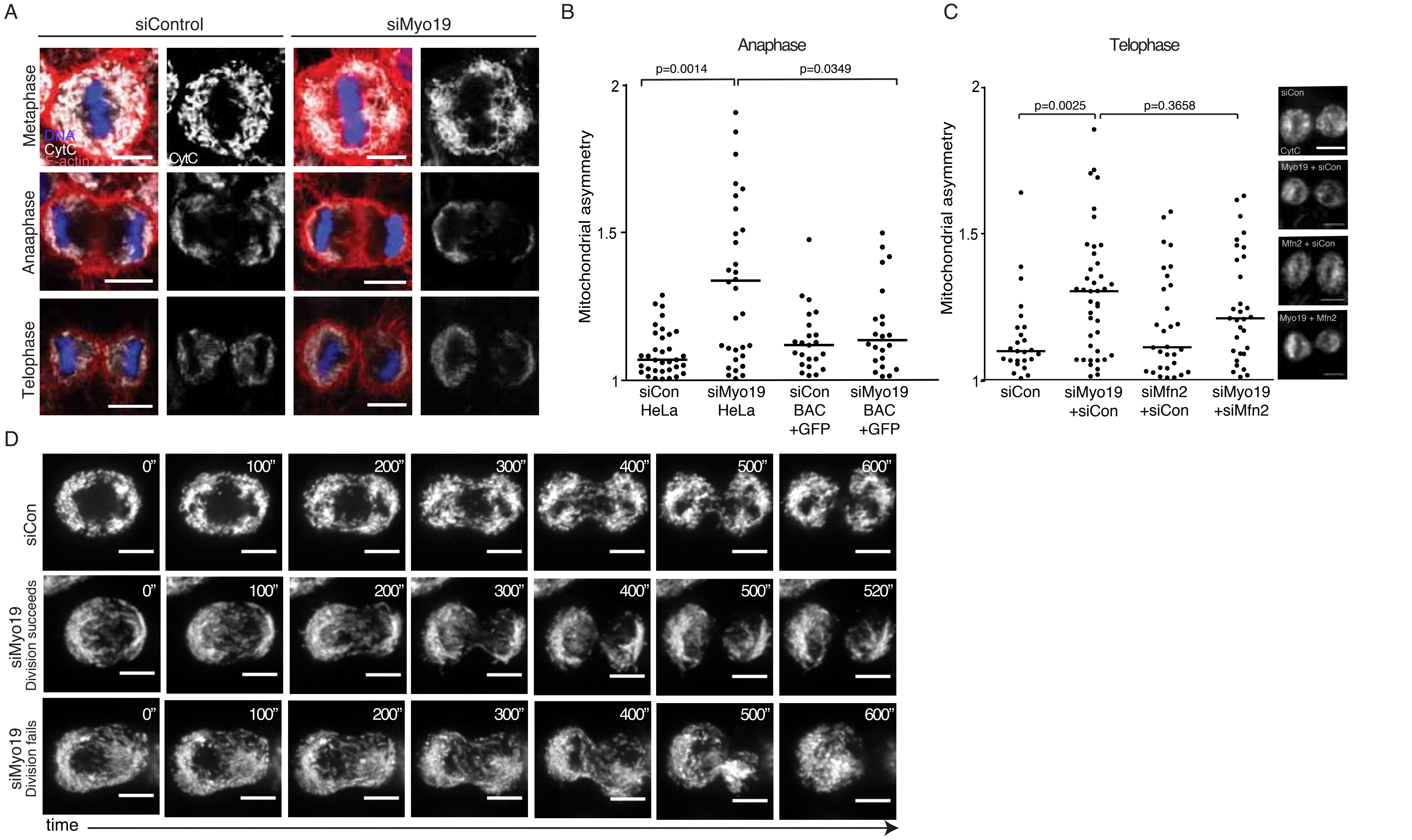


**Supplementary Figure S3.** Related to Figure 3. Myo19 depletion causes an asymmetric localization of mitochondria in multiple phases of cell division. (A) Cells treated with siControl or siMyo19 for 65 hr as indicated, at various stages of the cell cycle, fixed and stained with DAPI (blue), F-actin (red), and α-cytoch rome C (grey); greyscale counterparts are cytochrome C only. One plane through cell centre is shown. (B) Quantitation of mitochondrial asymmetry in anaphase of HeLa cells (left) or Myo19 BAC cells (right) treated with siCon or siMyo19 as indicated; the median is shown of at least 24 cells scored per condition. A number greater than 1 indicates relative asymme - try. (C) Analysis as for B but in HeLa telophase cells with the indicated siRNAs, scored in three different experiments. Insets to right show representative images (cytochrome C). (D) Timelapse imaging (every 10 sec) of HeLa cells treated with siRNA as indicated, labelled with MitoTracker Green 48 hr post-transfection. Images are maximum projections. Timestamps in seconds are indicated, with zero set during onset of anaphase. Scale bars are all 10 µm.

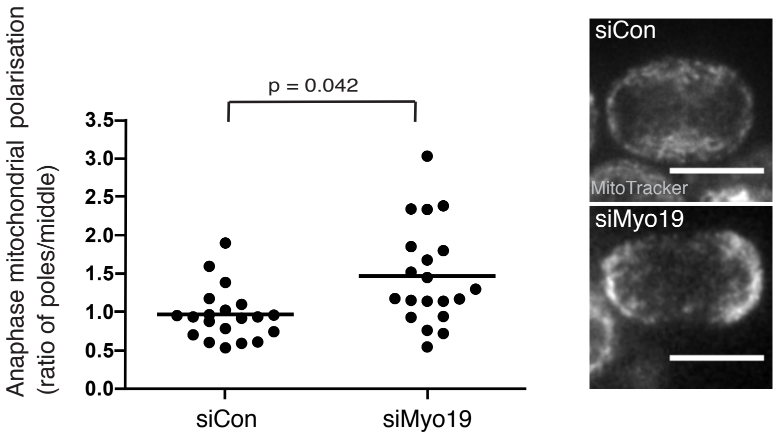


**Supplementary Figure S4.** Related to Figure 4. Lack of Myo19 results in mitochondrial accumulation at the spindle poles in anaphase. Quantification of mitochondrial presence at the poles of anaphase HeLa cells. This, like Figure 4A, phenocopies the effect of Latrunulin B treatment (as seen in Figure 4B). Movies were analysed in “total sum” mode to capture pixels in all planes, as in Figure 4C. A number greater than 1 indicates polar enrichment. On the right, representative images show Mitotracker Green labelling, Bar = 16 µm.

-

| Gene Name | siRNA Name | Plate No. | Spot position | Replicates analyzed [d] | Sense Sequence | NCBI Accession No. | Catalogue No. | Mean % Defect[a,e] | StDev[f] |
|---|---|---|---|---|---|---|---|---|---|
| ANLN | ANLN-01 | 1 | 366 | 1,2 | GGAGAUGGAUCAAGCAUUA | NM_018685 | D-006838-01 | 85 | 9 |
| ANLN | ANLN-02 | 1 | 79 | NA [c] | GAAAUCCGCUUGCCUCUAA | NM_018685 | D-006838-02 | | |
| ANLN | ANLN-03 | 1 | 175 | NP [b] | GGAUAAAUCUGGCUAAUUG | NM_018685 | D-006838-03 | | |
| ANLN | ANLN-05 | 1 | 270 | 1,3 | ACGCAACACUUUUGAAUUA | NM_018685 | D-006838-05 | 71 | 1 |
| BCR | BCR-02 | 5 | 206 | NA | GUAAAGCUCUCGGUCAAGU | NM_004327 | D-003875-02 | 21 | 1 |
| BCR | BCR-03 | 5 | 302 | 1,2 | GCAUUCCGCUGACCAUCAA | NM_004327 | D-003875-03 | 14 | |
| BCR | BCR-06 | 5 | 218 | 1 | CAGGAGCGCUUCCGCAUGA | NM_004327 | D-003875-06 | 15 | |
| BCR | BCR-13 | 5 | 314 | 1,2 | CAGAAGAAGUGUUUCAGAA | NM_004327 | D-003875-13 | | |
| CIT | CIT-01 | 1 | 244 | 1,3 | GAUAUUAGAUGCCCUCUUU | NM_007174 | D-004613-01 | 53 | 8 |
| CIT | CIT-02 | 1 | 340 | 1,2 | GGACCAGUCUUCAGUAUAA | NM_007174 | D-004613-02 | 63 | 2 |
| CIT | CIT-03 | 5 | 61 | NA | GGACAUCUAUGCUAUGAAA | NM_007174 | D-004613-03 | | |
| CIT | CIT-04 | 5 | 157 | NA | GGAGCAGUCUUCCAAAUUU | NM_007174 | D-004613-04 | | |
| ECT2 | ECT2-01 | 6 | 195 | 1,3 | GAUAAAGGAUGAUCUUGAA | NM_018098 | D-006450-01 | 45 | 12 |
| ECT2 | ECT2-02 | 6 | 291 | 1,3 | GCACUCACCUUGUAGUUGA | NM_018098 | D-006450-02 | 68 | 26 |
| ECT2 | ECT2-03 | 6 | 207 | NP | GAAGGGCUCUUAUGACAUC | NM_018098 | D-006450-03 | | |
| ECT2 | ECT2-04 | 6 | 303 | NA | CAGAGGAGAUUAAGACUAU | NM_018098 | D-006450-04 | | |
| MYO19 | MYOHD1-01 | 3 | 226 | NP | CGAUACAAGUUACUAAGAA | NM_025109 | D-017137-01 | | |
| MYO19 | MYOHD1-02 | 3 | 322 | 1,2,3 | GGACCAAGGUGUUCAUGAC | NM_025109 | D-017137-02 | 25 | 11 |
| MYO19 | MYOHD1-03 | 3 | 238 | 1,2,3 | CAAUACGGCUAUGGGUGUA | NM_025109 | D-017137-03 | 17 | 3 |
| MYO19 | MYOHD1-04 | 3 | 334 | 1,2,3 | UCAGGGAUCGAUAAAGUUU | NM_025109 | D-017137-04 | | |
| RACGAP1 | RACGAP1-01 | 6 | 68 | 1,3 | CAAAUUAUCUCUGAAGUGU | NM_013277 | D-008650-01 | 41 | 13 |
| RACGAP1 | RACGAP1-02 | 6 | 164 | NP | CCACAGACACCAGAUAUUA | NM_013277 | D-008650-02 | | |
| RACGAP1 | RACGAP1-03 | 6 | 260 | 1,3 | GAACAUCAGCUUCUCAAGA | NM_013277 | D-008650-03 | 27 | 7 |
| RACGAP1 | RACGAP1-04 | 6 | 356 | 1,3 | GUAAUCAGGUGGAUGUAGA | NM_013277 | D-008650-04 | 22 | 3 |
| SPTBN1 | SPTBN1-01 | 4 | 353 | 1,2,3 | CGGAAGAGAUCGCCAAUUA | NM_003128 | D-018149-01 | 25 | 16 |
| SPTBN1 | SPTBN1-02 | 4 | 65 | NP | GACGAGAUCUUGUGGGUUG | NM_003128 | D-018149-02 | | |
| SPTBN1 | SPTBN1-03 | 4 | 161 | NP | CUUAUGUGGUGACUUAUUA | NM_003128 | D-018149-03 | | |
| SPTBN1 | SPTBN1-04 | 4 | 257 | 1,2,3 | CGAGUGCAAUGAAACCAAA | NM_003128 | D-018149-04 | 14 | 5 |
| SPTBN5 | SPTBN5-01 | 4 | 271 | NP | GCACAGACACCAGGACUUA | NM_016642 | D-020903-01 | | |
| SPTBN5 | SPTBN5-02 | 4 | 367 | 1 | GGAAAGAUCUGGAGAGCGU | NM_016642 | D-020903-02 | 16 | |
| SPTBN5 | SPTBN5-03 | 4 | 283 | 1,2,3 | GCAGAAGUUUGGCCGAGAA | NM_016642 | D-020903-03 | 20 | 7 |
| SPTBN5 | SPTBN5-04 | 4 | 379 | 1 | CAACUGAGGCAGAGAUAUC | NM_016642 | D-020903-04 | 15 | |
| TPM4 | TPM4-01 | 4 | 244 | NP | CCAAGCACAUUGCGGAAGA | NM_003290 | D-019753-01 | | |
| TPM4 | TPM4-02 | 4 | 340 | 1,2,3 | GCGGAGGUGUCUGAACUAA | NM_003290 | D-019753-02 | 17 | 3 |
| TPM4 | TPM4-03 | 4 | 61 | NP | GAACGUGGGCUUACAUCAG | NM_003290 | D-019753-03 | 12 | |
| TPM4 | TPM4-04 | 4 | 157 | 1 | GGAUCAGACACUAAACGAA | NM_003290 | D-019753-04 | | |

| | | | | | | | | | |
|---|---|---|---|---|---|---|---|---|---|
| siCONTROL | OnTarget Plus non-targeting | 1 | 131 | 1 | Pool [g] | N/A | D-001810-01-05 | 6 [e] | 1 |
| siCONTROL | OnTarget Plus non-targeting | 1 | 145 | 1 | Pool [g] | N/A | D-001810-01-05 | | |
| siCONTROL | OnTarget Plus non-targeting | 1 | 179 | 1 | Pool [g] | N/A | D-001810-01-05 | | |
| siCONTROL | OnTarget Plus non-targeting | 3 | 190 | 1 | Pool [g] | N/A | D-001810-01-05 | 7 | 3 |
| siCONTROL | OnTarget Plus non-targeting | 3 | 179 | 1 | Pool [g] | N/A | D-001810-01-05 | | |
| siCONTROL | OnTarget Plus non-targeting | 3 | 145 | 1 | Pool [g] | N/A | D-001810-01-05 | | |
| siCONTROL | OnTarget Plus non-targeting | 4 | 145 | 1 | Pool [g] | N/A | D-001810-01-05 | 9 | 3 |
| siCONTROL | OnTarget Plus non-targeting | 4 | 190 | 1 | Pool [g] | N/A | D-001810-01-05 | | |
| siCONTROL | OnTarget Plus non-targeting | 4 | 179 | 1 | Pool [g] | N/A | D-001810-01-05 | | |
| siCONTROL | OnTarget Plus non-targeting | 4 | 148 | 1 | Pool [g] | N/A | D-001810-01-05 | | |
| siCONTROL | OnTarget Plus non-targeting | 4 | 142 | 1 | Pool [g] | N/A | D-001810-01-05 | | |
| siCONTROL | OnTarget Plus non-targeting | 4 | 139 | 1 | Pool [g] | N/A | D-001810-01-05 | | |
| siCONTROL | OnTarget Plus non-targeting | 4 | 100 | 1 | Pool [g] | N/A | D-001810-01-05 | | |
| siCONTROL | OnTarget Plus non-targeting | 5 | 97 | 1 | Pool [g] | N/A | D-001810-01-05 | 7 | 2 |
| siCONTROL | OnTarget Plus non-targeting | 5 | 142 | 1 | Pool [g] | N/A | D-001810-01-05 | | |
| siCONTROL | OnTarget Plus non-targeting | 5 | 179 | 1 | Pool [g] | N/A | D-001810-01-05 | | |
| siCONTROL | OnTarget Plus non-targeting | 6 | 100 | 1 | Pool [g] | N/A | D-001810-01-05 | 13 | 5 |
| siCONTROL | OnTarget Plus non-targeting | 6 | 142 | 1 | Pool [g] | N/A | D-001810-01-05 | | |
| siCONTROL | OnTarget Plus non-targeting | 6 | 148 | 1 | Pool [g] | N/A | D-001810-01-05 | | |

[a] Based on starting multinucleation rates plus accumulated defects; see Experimental Procedures for details
[b] NP = No multinuclear phenotype in fixed screen
[c] NA = Not analyzed as phenotype very strong with known gene; results for two siRNAs deemed sufficient
[d] For genes, <3 replicates analyzed when it was not possible to score accurately due to quality control issues (focus drift or initial overcrowding)
[e] For siControl, different spots on same plate were analyzed, not replicates per spot
[f] No standard deviations are available in cases where only one film could be analyzed due to quality control issues
[g] The siCONTROL is a pool of four non-targeting sequences: UGGUUUACAUGUCGACUAA, UGGUUUACAUGUUGUGUGA, UGGUUUACAUGUUUUCUGA, and UGGUUUACAUGUUUUCCUA

**Supplemental Table S1.** Related to Figure 1. Details of individual siRNAs pooled in Figure 1, including their cell multinucleation phenotypes.

**Supplemental Experimental Procedures**

**Cell culture.** HeLa-Kyoto human carcinoma cells and HeLa-Kyoto cells stably expressing histome-2B-mCherry [S1] (a kind gift from M. Petronczki) were cultured at 37°C in a humidified incubator under 5% $CO_2$ in Dulbecco's Modified Eagle Medium (DMEM, Gibco 41965) supplemented with 10% fetal bovine serum (FBS) (PAA) and antibiotics (50 U/ml penicillin and 50 μg/ml streptomycin; Gibco 15070). HeLa cells stably expressing LifeAct-GFP/histone2B-mRFP (Hela-13) [S2] were grown the same except with the addition of puromycin (Sigma) to 0.5 μg/ml and G418 (Gibco) to 500 μg/ml to maintain selection of the transgenes. HeLa cells expressing mouse GFP-Myo19 (Myo19-BAC), a kind gift from A. Hyman, were cultured as above except the medium contained 400μg/ml G418. The murine neuronal tumor cell line CAD [S3] was cultured at 37°C in a humidified incubator under 5% CO2 in DMEM/F12 supplemented with 10% fetal bovine serum and antibiotics. shRNA stable cell lines were maintained in the same medium supplemented with 2μg/mL puromycin. To derive cells with red-fluorescent microtubules, we used FuGene HD (Promega) with a tubulin-mCherry plasmid. To establish HeLa cells stably expressing mitochondria-targeted TagYFP (HeLa-Mito-YFP), we transfected the Mito-TagYFP plasmid (a kind gift from Phong Tran) and derived single-cell clones using standard methods after selection in G418 (500μg/ml).

**Antibody generation.** Rabbit anti-mouse Myo19 antibodies were raised against amino acids 814-963 of the mouse Myo19 protein expressed in *E. coli* as a 6xHis fusion protein from the pQE30 vector. Purified protein was used as the immunogen and sent to Zymed (Invitrogen) for injection into rabbits. Anti-Myo19 antibodies were purified from rabbit serum using a Myo19 affinity resin generated from the same expressed protein.

**siRNA library, live imaging screen and visual analysis.** A custom Dharmacon siRNA library targeting 515 cytoskeletal genes and actin regulatory genes, four individual siRNAs per gene, was previously described, including the morphological phenotypes of their silencing in a fixed immunofluorescence assay [S4]. We arrayed siRNA spots using a VersArray chipwriter (Bio-Rad) in a “transfection-ready” format onto LabTek chamber slides in triplicate (384 spots per slide, total of 8 slides) exactly as previously described [S5]. Before live imaging, the grid was oriented with pen marks and seeded with HeLa13 cells. Two days after solid-phase transfection, each spot on the slide was imaged automatically on an Olympus automated epifluorescence microscope (IX-81; modified as described in [S5]) using the 10x objective, binning 1, every 33 minutes for 20 hours, using two channels (green; F-

actin and red; nucleus) per timepoint. We used the data from our previous fixed screen [S4] to focus on genes (N=67) whose knockdown had produced a multinucleated cell phenotype. We narrowed this list to the 18 most robust hits. Replicate films of these were analysed frame-by-frame and various aspects quantitated. First we scored multinucleation at the start of the film, to take into account phenotypes that were already well advanced by the start of filming. Then we scored the division outcome (multinucleation or normal) of at least 100 cells per film that contained a single nucleus at the start of the film and appeared otherwise normal. This cumulative multinucleation rate was added to the rate of multinucleation at the start of the film to generate a net percentage defect. The results for each independent siRNA targeting the same gene that scored positively in the screen were pooled to generate a mean multinucleation rate per gene (see Supplemental Table S1 for the phenotypes of individual siRNAs). Each slide of 384 spots contained at least three siControl siRNA spots, which were analysed and pooled. Using this approach, we identified candidates that exhibited division defects, with more than one independent siRNA, more frequently than was evident in cells treated with the siControl reagent. Depending on the siRNA, divisions failed at a variety of stages, from early anaphase all the way through to multinucleation resulting from daughter cell re-fusion during interphase.

**Smaller-scale siRNA experiments.** All siRNA experiments in HeLa and BAC cells were performed using reverse transfection with Lipfectamine-2000 (Life Technologies) according to the manufacturer's instructions, with Dharmcon siGenome duplexes (GE Healthcare). We tested the following siGenome siRNAs from the same company: Myo19-01, -02, -03, -04, -10, -11 and -12. Myo19-03 was used as the reagent once we had established that multiple siRNAs gave the same phenotypes (see the Results section for details; of this set, our trials indicated that only Myo19-01 and Myo19-12 had no phenotype). We also used an siRNA against mitofusion-2 (J-012961-07; sequence GCAACUCUAUCGUCACAGU). Our negative control was the OnTarget Plus control non-targeting siRNA Pool (Dharmacon/GE Healthcare, D-001810-01-05, referred to throughout the paper as siControl). We used the following conditions for transient transfection of HeLa and BAC cells: for 384-well black, thin-bottomed confocal-ready tissue-culture plates (Greiner bio-one): final volume of 60μL (final siRNA concentration of 25 nM) per well and 1000 cells; for 3 cm glass-bottomed dishes and 12-well glass-bottomed plates (Mattek): final volume of 300μL (final siRNA concentration of 20nM, containing 15,000 cells) per well, and topped up to 2mL after 6-8 hours. For transfections involving two siRNAs, the total amount of RNA was doubled and when compared with a single siRNA, the total amount was kept constant by the addition of an equal amount of siControl.

For experiments involving stable knockdown of Myo19, a MISSION plasmid for silencing the mouse Myo19 gene was purchased from Sigma (TRCN0000110628). To generate virus, HEK293FT cells were plated in T75 flasks at a concentration of approximately 5 million cells per plate and allowed to adhere overnight. Cells were then transfected with 1.25μg shRNA plasmid, 1.25μg PRRE plasmid, 1.25μg VSVG plasmid, 1.25μg REV plasmid (AddGene), and 0.2μg GFP-C1 plasmid (to verify transfection) using Fugene HD transfection reagent (Promega). After 24h the media in the flask was changed with 12ml fresh media. 24h later, the media was collected, and spun at 3000xg for 10 minutes. The supernatant was then filtered through a 0.45um filter and brought to 15ml. To infect CAD cells, a T25 flask that was ~70% confluent was treated with 3ml of virus-containing media supplemented with 8ug/ml hexamethadine bromide, and allowed to grow for two days. To select for stable incorporation of the plasmid DNA into the CAD cell genome, cells were then maintained in media containing 2μg/ml puromycin until a mixed population of cells with decreased Myo19 expression arose. Myo19 knockdown was verified via standard Western blotting using the polyclonal rabbit α-mouse Myo19 antibody at 0.17ug/ml overnight in TBS-0.1% Tween-20, or α-tubulin antibody (DM1A, Sigma) as a loading control, followed by the appropriate HRP-conjugated α-IgG antibodies and development using BioRad Clarity. Aside from its obvious strong and penetrant visual phenotype, Mfn2 depletion was assayed via standard Western blotting using a polyclonal rabbit antibody (H-68, sc-50331, Santa Cruz Biotechnology) diluted to 1:200; partial depletion was observed. The fact that virtually all mitochondria were fragmented after Mfn2 siRNA treatment suggests that expression of the family member Mfn1 cannot functionally compensate for even incomplete Mfn2 depletion in HeLa cells.

**Quantitative PCR**

Three days following siRNA treatments, we isolated RNA using QIAShredder and RNeasy (Qiagen) and prepared cDNA using a cDNA synthesis kit (Invitrogen), all according to the manufacturer's instructions. For quantitative PCR, we performed each reaction in triplicate using 150 ng DNA using the DyNAmo Flash SYBR Green qPCR kit (Qiagen). Primers used were: Myo19 Forward, 5' GGA CAA CCA GCC CTG TTT GGA TCT ; Myo19 Reverse, 5' TGG TCA GCT CAG GTG GGA TAG GGT; controls included standard primers for actin (a kind gift from Dan Cutler; Actin Forward 5' TGG TGG TGA AGC TGT AGCC 3'; Actin reverse 5' GCG AGA AGA TGA CCC AGAT 3') and reactions in which DNA was not included. Experiments using GAPDH as the standardization gene gave similar results (data not shown). Data were normalised by the delta-delta-CT method.

**Cell staining and microscopy.** For HeLa and BAC cell experiments, cells were fixed with freshly prepared formaldehyde (4% in PBS) for 20 mins at RT, permeablized with 0.2% Triton-X in PBS for 5 minutes and blocked with 5% BSA in PBS for 30 minutes. Depending on the experiment, we stained with TRITC-conjugated phalloidin (0.125μg/ml, Sigma P1951); DAPI (1 μg/ml, Sigma D9564), α-GFP (1:200; Invitrogen) and/or α-cytochrome C (1:200; Abcam) in a PBS solution containing 1% BSA for 1 hr. For antibodies, we used the appropriate fluorescently coupled Alexa-Fluor-α-IgG secondary antibodies from Molecular Probes at 1:500 for a further hour. After 3x washing with PBS, plates were stored in PBS containing 0.1% sodium azide (Sigma). Fixed images were acquired using a Leica SP5 or SPE3 scanning confocal microscope. All images were processed according to good practice using ImageJ/FIJI [S6].

For CAD experiments, cells were stained with 100nM Mitotracker Red CMXRos (Life Technologies) in DMEM for 15 minutes, followed by 2x 10-minute washes in DMEM and a quick rinse in PBS. Cells were then fixed in 4% paraformaldehyde (Electron Microscopy Sciences) in PBS for 10 minutes, permeabilized in PBS with 0.5% TritonX-100 (Sigma) for 5 minutes, and then stained with 75 nM DAPI and 7nM alexa-488-phalloidin (Molecular Probes) for 15 minutes. Coverslips were mounted in PBS containing 80% glycerol containing 0.5% N-propyl gallate. Cells were examined using a Olympus fluorescence microscope with a 60x/1.4NA and 40x/1.3NA objective with an attached Orca FLASH4v2 cooled CMOS digital camera (Hamamatsu) managed with Metamorph software, or a Nikon fluorescence microscope with a 60x/1.4NA objective with an attached Coolsnap HQ2 cooled CCD digital camera (Photometrics) managed by μManager [S7]. Images were analyzed using Metamorph or Fiji [S6].

**Live imaging.** For high-resolution live imaging of mitochondria, HeLa cells were transfected for 24 hours, after which MitoTracker Green (0.1 μM, Molecular Probes) was added for 30 mins before filming with the imaging medium (Fluorobrite DMEM [Gibco] + 10% FBS with the normal antibiotics as above). Cells were washed with PBS then imaged on an UltraVIEW VoX spinning disk confocal microscope (PerkinElmer) with the 60x objective lens every 20 sec (Supplemental Figure S3D) or every 2 min (Supplemental Figures S4 and Figure 4), or in the case of Supplemental Figure 4A, overnight filming with 3 min intervals and 2 μm sections. Cells for Figure 1B-C and Supplemental Figures S2B and D were prepared similarly except that imaging was conducted on an Axiovert 200M (Zeiss) with a motorized stage. For experiments involving synchronization at prometaphase, we used S-trityl-L-

cysteine (STLC; Sigma) at 5 μM treatment for 15 hr. When these experiments required additional manipulations, they were as follows: siRNAs were transfected 1 day before STLC addition, and plasmid transfected 12 hours before STLC addition. Next, MitoTracker Green was added for 30 mins, after which cells washed 5X with the imaging medium before filming. For the actin inhibition experiment after washing out the MitoTracker, we started filming, then added Latrunculin B (to 5 μM) or an equal volume of DMSO and resumed filming 14 minutes later at the same x/y point. Two hours later, we fixed and stained the cells as for HeLA and BACS, above, to confirm that the F-actin network had been completely dismantled by the Latrunculin treatment.

**Quantitation of mitochondrial symmetry and spindle pole localization.** In confocal images of dividing cells, Z-slices were summed for total pixel intensity in the channel corresponding to cytochrome C. Using ImageJ, two identical polygons were drawn to measure mean intensity, which was then multiplied by the area to get the total. Background intensity was subtracted, and the larger amount was divided by the smaller amount to obtain the mitochondrial ratio. A ratio of 1 would indicate symmetry, whereas higher amounts indicate asymmetry. To determine the ratio of mitochondria at spindle poles compared with the cell middle during anaphase, we used ImageJ to draw two identically sized polygons at each pole (cells viewed as "total sum"), to harvest all pixel information from all the stacks, and drew a third contiguous polygon in the middle that was exactly twice the size of each polar polygon. We measured mean intensity in all three polygons, multiplied this by each corresponding area to achieve the total, and then background intensity was subtracted. Then we added the pole numbers together and divided this number by the number for the middle polygon to obtain the spindle pole/middle ratio. A ratio greater than 1 would indicate an accumulation of mitochondria at the spindle poles.

**Statistics.** All statistics were performed using PRISM (GrafPad Software) or MiniCAD (Vectorworks).

## Supplemental References


S1. Su, K.C., Takaki, T., and Petronczki, M. (2011). Targeting of the RhoGEF Ect2 to the equatorial membrane controls cleavage furrow formation during cytokinesis. Developmental cell *21*, 1104-1115.

S2. Matthews, H.K., Delabre, U., Rohn, J.L., Guck, J., Kunda, P., and Baum, B. (2012). Changes in Ect2 localization couple actomyosin-dependent cell shape changes to mitotic progression. Developmental cell *23*, 371-383.

S3. Qi, Y., Wang, J.K., McMillian, M., and Chikaraishi, D.M. (1997). Characterization of a CNS cell line, CAD, in which morphological differentiation is initiated by serum deprivation. J Neurosci *17*, 1217-1225.

S4. Rohn, J.L., Sims, D., Liu, T., Fedorova, M., Schock, F., Dopie, J., Vartiainen, M.K., Kiger, A.A., Perrimon, N., and Baum, B. (2011). Comparative RNAi screening identifies a conserved core metazoan actinome by phenotype. J Cell Biol *194*, 789-805.

S5. Neumann, B., Held, M., Liebel, U., Erfle, H., Rogers, P., Pepperkok, R., and Ellenberg, J. (2006). High-throughput RNAi screening by time-lapse imaging of live human cells. Nature methods *3*, 385-390.

S6. Schindelin, J., Arganda-Carreras, I., Frise, E., Kaynig, V., Longair, M., Pietzsch, T., Preibisch, S., Rueden, C., Saalfeld, S., Schmid, B., et al. (2012). Fiji: an open-source platform for biological-image analysis. Nature methods *9*, 676-682.

S7. Edelstein, A., Amodaj, N., Hoover, K., Vale, R., and Stuurman, N. (2010). Computer control of microscopes using microManager. Current protocols in molecular biology / edited by Frederick M. Ausubel ... [et al.] *Chapter 14*, Unit14 20.
